# Arginine methylation promotes siRNA-binding specificity for a spermatogenesis-specific isoform of the Argonaute protein CSR-1

**DOI:** 10.1038/s41467-021-24526-6

**Published:** 2021-07-09

**Authors:** Dieu An H. Nguyen, Carolyn M. Phillips

**Affiliations:** Department of Biological Sciences, University of Southern California, Los Angeles, CA USA

**Keywords:** siRNAs, Methylation, Alternative splicing

## Abstract

CSR-1 is an essential Argonaute protein that binds to a subclass of 22G-RNAs targeting most germline-expressed genes. Here we show that the two isoforms of CSR-1 have distinct expression patterns; CSR-1B is ubiquitously expressed throughout the germline and during all stages of development while CSR-1A expression is restricted to germ cells undergoing spermatogenesis. Furthermore, CSR-1A associates preferentially with 22G-RNAs mapping to spermatogenesis-specific genes whereas CSR-1B-bound small RNAs map predominantly to oogenesis-specific genes. Interestingly, the exon unique to CSR-1A contains multiple dimethylarginine modifications, which are necessary for the preferential binding of CSR-1A to spermatogenesis-specific 22G-RNAs. Thus, we have discovered a regulatory mechanism for *C. elegans* Argonaute proteins that allows for specificity of small RNA binding between similar Argonaute proteins with overlapping temporal and spatial localization.

## Abstract (web summary)

The Argonaute protein CSR-1 is essential for fertility and viability in *C. elegans*. Here the authors show that CSR-1A isoform associates preferentially with small RNAs mapping to spermatogenesis-specific genes while CSR-1B isoform binds small RNAs mapping to oogenesis-specific genes. Arginine methylation of CSR-1A promotes small RNA-binding specificity.

## Introduction

In the race for evolutionary fitness, sexual selection is a strong selective force. Multicellular eukaryotes have long favored sexual divergence of the male and female genders to increase genetic diversity, often resulting in sexual dimorphism. In *C. elegans*, a protandrous nematode, sexual reproduction can occur in a single germline where spermatogenesis and oogenesis take place sequentially. Both eggs and sperm are derived from the same pool of mitotically replicating nuclei at the distal tip of each gonad arm, which subsequently undergo meiosis as they progress through the germline toward the proximal end. During the last larval stage (L4), ~40 meiotic nuclei in each gonad arm begin the process of spermatogenesis, where they differentiate into mature sperm^1^. Subsequently, oogenesis begins as the animal enters the adult stage, and the germline continues to produce oocytes for the rest of the reproductive cycle^2^.

Any given germ cell has the potential to differentiate into either spermatocytes or oocytes, though once committed to a sexual fate, the cells undergo distinct meiotic programs that vary remarkably in rate and mechanics^3^. Regardless of these differences, meiosis occurs exclusively in germ cells, which are surrounded by an environment inducive to the expression of germline-specific transcripts and suppression of somatic transcripts. One mechanism by which *C. elegans* germ cells promote this selective environment is by way of a perinuclear structure called the P granule^4^. These germline granules, which help to ensure pluripotency and germ cell identity, form a phase-separated condensate outside of the nucleus and contiguous with nuclear pores, where they regulate newly synthesized germline transcripts^4–7^. One key regulator of P granule morphology and *C. elegans* germline development is the Argonaute protein, CSR-1 (Chromosome Segregation and RNAi deficient)^8,9^.

Argonaute proteins are the core effectors of all small RNA-related pathways. These proteins bind small guide RNAs and regulate complementary transcripts by either directly cleaving target RNAs or recruiting cofactors that promote transcriptional or posttranscriptional gene regulation^10,11^. Of the ~27 Argonaute proteins annotated in the *C. elegans* genome, only CSR-1 is essential for fertility, and it is reported to regulate over 4000 germline-expressed genes^8,12^. CSR-1 binds to a class of antisense 22-nucleotide small interfering RNAs with a 5′ guanosine (22G-RNAs) whose target transcripts are thought to be protected from silencing by other small RNA-mediated pathways, and are thus “licensed” for germline expression^8,13,14^. In contrast, foreign DNA such as transposons and transgenes containing non-*C. elegans* sequences are recognized by piwi-interacting RNAs (piRNAs, also known as 21U-RNAs in *C. elegans*), which, along with their Argonaute cofactor PRG-1, can initiate a multigenerational epigenetic silencing signal that depends on chromatin factors and 22G-RNAs bound by the worm-specific Argonaute proteins (WAGOs)^15–17^. The balance of gene licensing and gene silencing by CSR-1 and the WAGO proteins, respectively, is critical to promote expression of essential germline genes; and a disrupted balance severely compromises germline development and fertility^18,19^.

Studies of CSR-1 have reported several severe phenotypes that lead to defects in viability and fertility. In *csr-1* mutant embryos, chromosomes fail to properly align on the metaphase plate, resulting in aberrant mitotic division and anaphase bridging^8,12,20^. In the adult germline, loss of CSR-1 leads to fewer germ cells, defects in meiotic progression, and a delayed spermatogenesis to oogenesis switch^21^. Additionally, CSR-1 has been implicated in a myriad of other cellular processes, including maturation of core histone mRNAs, promotion of sense-oriented RNA polymerase II transcription, attenuation of translation elongation, prevention of premature activation of embryonic transcripts in oocytes, alternative splicing, and paternal inheritance^22–27^. Yet how one Argonaute protein can regulate so many seemingly distinct processes remains unanswered.

Here we demonstrate that CSR-1 has two isoforms—CSR-1B, which is present throughout the germline and CSR-1A, which is specific to spermatogenic germ cells. CSR-1A and CSR-1B associate with distinct subsets of small RNAs to regulate spermatogenic or oogenic transcripts, respectively. The specificity of the two CSR-1 isoforms is interesting, considering they share nearly complete sequence homology and co-localize at the P granule in L4 larval and male germlines. We found that the first exon of CSR-1A is modified at arginine/glycine (RG) motifs by dimethylarginine. Here we show that loss of the dimethylarginine results in the loss of CSR-1A specificity for its preferred spermatogenic small RNA partners, resulting in CSR-1A indiscriminately binding to both spermatogenic and oogenic siRNAs. Thus, in this study, we have identified the first instance of methylarginine modification of a *C. elegans* Argonaute protein and demonstrated a previously unappreciated mechanism by which Argonaute proteins can acquire small RNA specificity.

## Results

### The long isoform of CSR-1 is selectively expressed during spermatogenesis

**Fig. 1:**
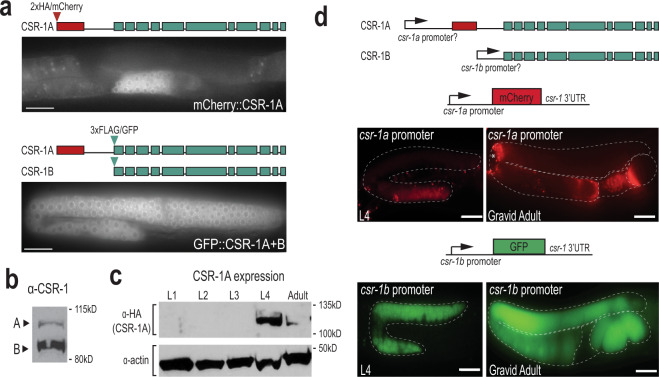
CSR-1 isoforms have distinct spatial and temporal expression patterns. **a** Live imaging of CSR-1A (top) and CSR-1A + B (bottom) in an L4 hermaphrodite germline. At least three individual germlines were imaged for each strain. Scale bars, 25 μM. **b** Western blot detecting CSR-1 expression, using α-CSR-1 antibodies, in wild-type animals at L4 stage. **c** Western blot detecting for CSR-1A at each larval stage and gravid adult in 2xHA::mCherry::CSR-1A strain using α-HA antibody. Actin is shown as a loading control. **d** Schematic representation (top) of putative *csr-1a* and *csr-1b* promoters driving mCherry and GFP. The *csr-1a* promoter comprises ~1.5 kb of sequence preceding the first *csr-1a* exon, and the *csr-1b* promoter is the entire 544-bp intron between the unique *csr-1a* exon and the start codon of *csr-1b*. Schematic of the promoter reporter constructs created using the MosSCI system for *csr-1a* (middle) and *csr-1b* (bottom) are shown above images of gonads from L4 and gravid adult stages expressing the reporter constructs. Asterisk indicates intestinal autofluorescence visible near the bend in adult *csr-1a::mCherry* gonad. At least three individual germlines were imaged for each strain and condition. Scale bars, 25 μM. All blots have been reproduced. Source data are provided as a Source data file.

The Argonaute protein, CSR-1, has two isoforms, though previously little was known about their distinct functions. The longer isoform, referred to as CSR-1A, and the shorter isoform, CSR-1B, share complete sequence homology, except for a unique exon at the 5′ end of CSR-1A (Fig. 1a). Both isoforms are expressed, though at differential levels (Fig. 1b)^8,20^. Furthermore, transcriptome analysis suggests that CSR-1A is expressed in spermatogenic gonads, but excluded from oogenic gonads^28^. To explore the distinct functions of the two CSR-1 isoforms, we used CRISPR/Cas9 to endogenously tag CSR-1A at its N-terminus with a 2xHA/mCherry tag. We also tagged both CSR-1A and CSR-1B in the same strain by adding a 3xFLAG/GFP tag to the N-terminus of CSR-1B (Fig. 1a). When both isoforms are tagged, CSR-1A + B is strongly expressed throughout the germline during both the fourth larval (L4) and adult stages, in agreement with previous studies (Fig. 1a)^8^. Interestingly, when only CSR-1A is tagged, expression is restricted to the spermatogenesis region of the germline in L4 hermaphrodites and males (Fig. 1a and Supplementary Fig. 1). We confirmed this observation by western blot analysis of all larval stage and adult animals, detecting CSR-1A expression robustly during the L4 stage and very weakly in gravid adults, coinciding temporally with spermatogenesis in *C. elegans* (Fig. 1c). Taken together, these results demonstrate that though both CSR-1 isoforms are expressed in the germline, CSR-1A expression is limited to germ cells undergoing spermatogenesis while CSR-1B is expressed throughout the germline in larval and adult animals.

### CSR-1A and CSR-1B are expressed from independent promoters

To address how CSR-1A and CSR-1B have such distinct expression patterns, we sought to determine whether they are expressed from independent promoters. We first fused ~1.5 kb of DNA preceding the *csr-1a* start codon to an mCherry reporter and exogenously expressed it in the germline using the MosSCI system (Fig. 1d)^29^. The region preceding the *csr-1a* start codon, the putative *csr-1a* promoter, drives mCherry expression only during the L4 stage and, during this time, expression is restricted to the spermatogenesis region of the germline. In gravid adults, residual mCherry expression can be detected inside the spermatheca, but is completely absent from the rest of the germline (Fig. 1d). To determine whether any regulatory sequences reside in the intron between the unique *csr-1a* exon and the start codon for *csr-1b*, we fused ~0.5 kb of DNA from this intron to a GFP reporter and expressed it in the germline using the MosSCI system (Fig. 1d). This region, the putative *csr-1b* promoter, drives GFP expression throughout the germline cytoplasm, in all developmental stages. The GFP expression can be observed in oocytes and fertilized embryos but is excluded from the spermatheca (Fig. 1d). These promoter fusion experiments corroborate our western blot analysis and indicate that CSR-1A is expressed predominantly during spermatogenesis, hinting at a unique role for CSR-1A during this developmental time point. Furthermore, the promoter fusion transgenes were able to recapitulate the expression pattern of the isoform-specific translation fusion proteins (Fig. 1a), indicating that the promoters are sufficient to drive the distinct expression patterns. Together, these data indicate that the intronic region that sits between the first *csr-1a* exon and the start codon for *csr-1b* contains the promoter sequence for CSR-1B, and that their distinct promoters independently establish the differential expression patterns of CSR-1A and CSR-1B.

### CSR-1A localizes to the P granules during spermatogenesis

**Fig. 2:**
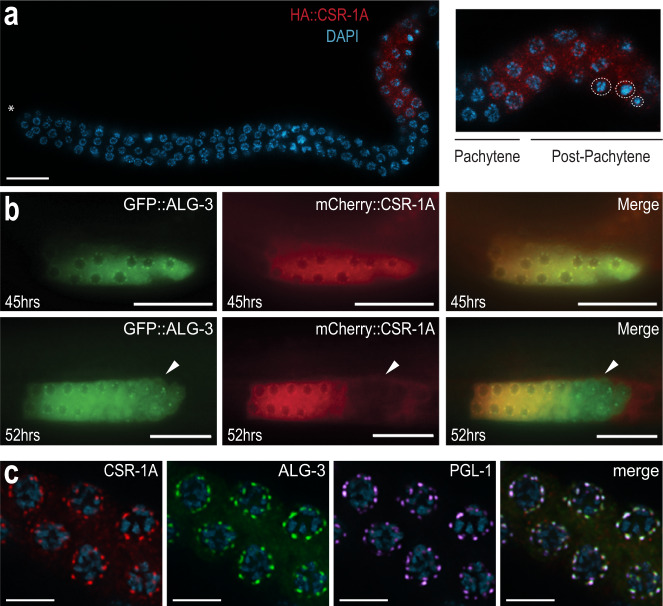
CSR-1A is expressed in the spermatogenesis region of the germline. **a** Immunofluorescence staining for HA::CSR-1A in dissected L4 hermaphrodite germline, using α-HA. Asterisk indicates distal end. Inset indicates region of cells exiting pachytene and entering post-pachytene where CSR-1A expression is detected. Dashed circles indicate karyosomes. DNA is stained with DAPI, blue. The experiment has been reproduced with at least five individual germlines imaged for each strain. Scale bar, 15 μM. **b** 3xFLAG::GFP::ALG-3 (left), 3xHA/mCherry::CSR-1A (middle), and merge (right) at 45-(early L4 stage) or 52-h (young adult stage) post-L1 arrest. White arrows indicate region of secondary spermatocytes. At least five individual germlines were imaged for each strain and condition. Scale bars, 25 μM. **c** Immunofluorescent staining of a 2xHA::mCherry::CSR-1A; GFP::3xFLAG::ALG-3 dissected L4 hermaphrodite germline, using antibodies against HA, FLAG, and PGL-1. The experiment has been reproduced with at least five individual germlines imaged for each strain. Scale bars, 5 μM.

To further characterize the expression pattern of CSR-1A, we immunostained for CSR-1A and DAPI-stained for germline nuclei in dissected L4 hermaphrodite gonads. As the germline progresses towards the proximal end, germ cells undergo early stages of meiosis up until pachytene in a non-sex-specific manner^3^. In post-pachytene, however, spermatocytes and oocytes commit to their respective sexual fates, and spermatocyte chromosomes exhibit an extensive condensation phase^3^. During L4, when spermatogenesis begins, CSR-1A expression is first observed in cells that are exiting pachytene, namely cells transitioning into diplotene and karyosome, indicating that CSR-1A is selectively present in spermatocytes (Fig. 2a). This expression is reminiscent of the sperm-specific Argonaute protein ALG-3, which is also present in spermatocytes during spermatogenesis^26^. To further investigate the role of CSR-1A during spermatogenesis, we carefully examined protein localization at different time points. As ALG-3 is another Argonaute protein that is expressed during spermatogenesis and is required for proper sperm development, we created a double-transgenic strain labeling both CSR-1A and ALG-3, using CRISPR. At early L4, or 45 h post hatching, both CSR-1A and ALG-3 form perinuclear foci in the spermatogenesis region that colocalize with the P granule marker PGL-1 (Fig. 2b, c). In contrast, CSR-1A + B forms perinuclear foci throughout the germline (Supplementary Fig. 2a). As the animals reach the L4 to adult transition, 52 h post hatching, CSR-1A and ALG-3 still colocalize during early spermatogenesis. However, by the time germ cells reach the secondary spermatocyte stage, CSR-1A expression becomes undetectable, while ALG-3 expression persists (Fig. 2b, lower panel, Supplementary Fig. 2b). PGL-1 similarly disappears from P granules in primary spermatocytes and is subsequently cleared from secondary spermatocytes^30^. Together, these data demonstrate that like ALG-3, CSR-1A localizes to P granules during spermatogenesis, but unlike ALG-3, CSR-1A expression is restricted to the early stages of spermatogenesis and is not found in secondary spermatocytes.

### CSR-1A is required for optimal sperm fertility

**Fig. 3:**
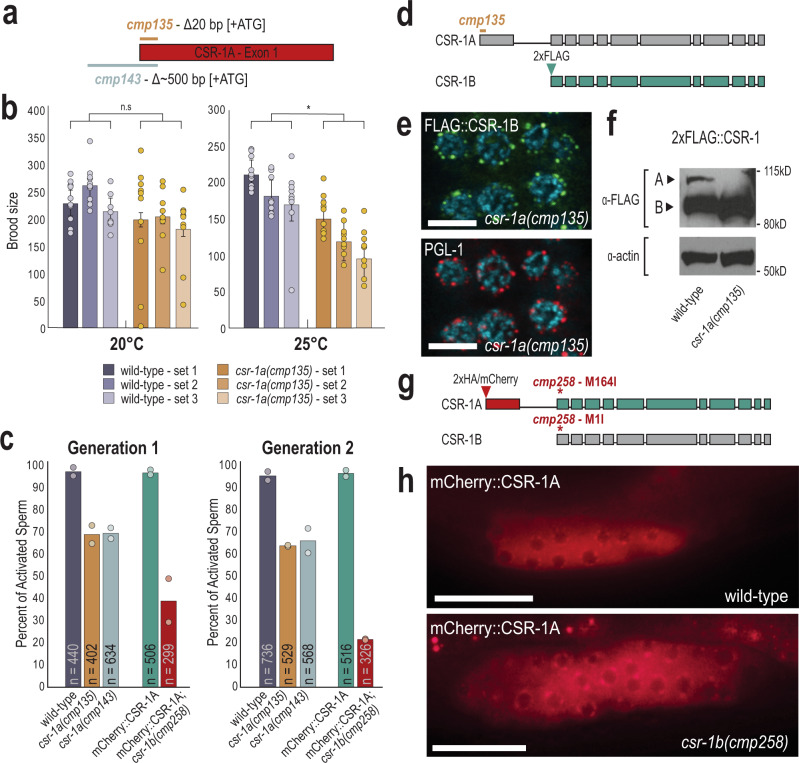
CSR-1 isoforms function independently of one another. **a** Schematic representation of two deletion alleles of *csr-1a*. *cmp135* removes 20 bp of coding sequence, including the start codon. *cmp143* removes ~500 bp of promoter sequence and 20 bp of coding sequence. **b** Brood-size assay performed on wild-type and *csr-1a* mutant animals at 20 and 25 °C. Three replicates were performed for each genotype, with ten animals per replicate. For each biological replicate, brood sizes for all ten animals are shown, with bar graphs representing mean brood size and error bars indicating standard deviation. Two-tail *t*-tests were performed to determine statistical significance. n.s. denotes not significant and indicates a *p* value >0.05 and * indicates a *p* value ≤0.05. See Supplementary Data 7 for more details regarding statistical analysis. **c** An in vitro sperm activation assay was performed on spermatids from wild-type, *csr-1a*(*cmp135*), *csr-1a*(*cmp143*), mCherry::CSR-1A, and mCherry::CSR-1A *csr-1b*(*cmp258*) mutants. Animals were raised at 25 °C for either one or two generations and at least 80 spermatids were counted for each replicate at each generation. Two biological replicates are shown, with bar graphs representing the mean. **d** Schematic representation of 2xFLAG::CSR-1B in a *csr-1a*(*cmp135*) mutant. The 2xFLAG tag was introduced immediately after the CSR-1B start codon in a *csr-1a*(*cmp135*) mutant animal. **e** Immunofluorescence staining for 2xFLAG::CSR-1B and P granules (PGL-1) in a *csr-1a*(*cmp135*) mutant at L4 stage. The experiment has been reproduced with at least five individual germlines imaged. Scale bar, 5 μM. **f** Western blot for CSR-1 expression levels, in wild-type and *csr-1a*(*cmp135*) mutant animals at L4 stage. Actin is shown as a loading control. This blot has been reproduced. **g** Schematic representation of disrupting *csr-1b* expression in a 2xHA/mCherry::CSR-1A strain. The *csr-1b* start codon was mutated from methionine to isoleucine (M1I) using CRISPR, which also introduces a M164I point mutation in the CSR-1A protein. **h** Live imaging of mCherry::CSR-1A in a wild-type and *csr-1b*(*cmp258*) mutant L4 hermaphrodite. At least five individual germlines were imaged for each strain. Scale bar, 25 μM. Source data are provided as a Source Data file.

We next sought to determine the role of CSR-1A in fertility. We first created two mutant alleles of *csr-1a* using CRISPR (Fig. 3a). *csr-1a*(*cmp135*) removes the first seven amino acids including the start codon and *csr-1a*(*cmp143*) removes the same region plus ~500 bp of the *csr-1a* promoter region. We confirmed that the mutants have severely reduced *csr-1a* expression via RT-qPCR and western blot analysis (Supplementary Fig. 3a, b). We first examined the fertility of *csr-1a* mutants by comparing the brood size of *csr-1a*(*cmp135*) hermaphrodites to wild-type hermaphrodites at 20 and 25 °C. We did not observe a significant reduction in brood size in *csr-1a*(*cmp135*) mutants compared to wild-type animals at 20 °C. However, at 25 °C, the brood size of *csr-1a*(*cmp135*) mutant animals was significantly reduced compared to wild-type animals (Fig. 3b). Because CSR-1A is expressed during spermatogenesis, we next sought to determine if CSR-1A is required for optimal male fertility.

One aspect of sperm quality that can be tested in vitro is sperm activation. Sperm activation is the process by which round spermatids are transformed into mature and motile spermatozoa^31^. This process can be recapitulated in vitro using Pronase E, a cocktail of proteases that induces sperm maturation and pseudopod formation^32^. Because *C. elegans* fertility is reduced at 25 °C, we raised wild-type and *csr-1a* mutant males at 25 °C to assess sperm activation under compromised conditions. Wild-type spermatids from males that were raised at 25 °C for one generation were activated at a rate of 97%, and after two generations at 25 °C, they were activated at a rate of 96% (Fig. 3c). In *csr-1a* males, we saw a modest reduction in the percentage of activated spermatids after one and two generations at 25 °C (69% for *cmp135* and 70% for *cmp143* after one generation, and 64% for *cmp135* and 66% for *cmp143* after two generations) (Fig. 3c). Because the effects we observed on sperm activation were modest yet significant, we generated two additional alleles of *csr-1a* to confirm our results. These new alleles remove the majority of the unique *csr-1a* exon (Supplementary Fig. 3c). Similar to what we observed with *csr-1a*(*cmp135*) and *csr-1a*(*cmp143*), the two new alleles, *csr-1a*(*cmp253*) and *csr-1a*(*cmp254*) reduced the percentage of activated spermatids by ~25–30% (Supplementary Fig. 3d). Together, these data suggest that CSR-1A contributes to optimizing fertility and is necessary to promote sperm activation.

### CSR-1A and CSR-1B are expressed independently of one another

Disruption of CSR-1 expression results in severe infertility and embryonic lethality^8,12^. To determine if our *csr-1a* alleles also affect the expression of CSR-1B protein, we introduced a 2xFLAG tag immediately following the start codon of CSR-1B in the *csr-1a*(*cmp135*) mutant background using CRISPR (Fig. 3d). Similar to wild-type, CSR-1B in the *csr-1a*(*cmp135*) mutant localized to perinuclear foci associated with the P granule marker, PGL-1 (Fig. 3e). Furthermore, by western blot, the CSR-1B protein was expressed in wild-type and *csr-1a*(*cmp135*) mutant animals, whereas the CSR-1A was undetectable in the *csr-1a*(*cmp135*) mutant but present in the wild-type strain (Fig. 3f). Interestingly, we observed that CSR-1B appears to accumulate at a modestly higher level in the *csr-1a*(*cmp135*) mutant, but not in the *csr-1a*(*cmp143*) mutant (Supplementary Fig. 3b). To determine if CSR-1B is required for CSR-1A expression, we generated an effectively null allele of *csr-1b*, where we mutated the *csr-1b* start codon to isoleucine, in the mCherry::CSR-1A strain using CRISPR (Fig. 3g). In this *csr-1b*(*cmp258*) mutant, mCherry::CSR-1A is expressed at perinuclear foci in the spermatogenesis region of L4 animals, similar to wild-type expression of CSR-1A (Fig. 3h). These animals, however, are sterile and need to be maintained over a balancer, a phenotype previously associated with *csr-1* mutants^12^.

Because CSR-1B is expressed during spermatogenesis and, like ALG-3, is present in the secondary spermatocytes, (Supplementary Fig. 2a, b), we next asked if CSR-1B contributes to optimizing sperm health. To this end, we performed in vitro sperm activation on *csr-1b*(*cmp258*) mutants at 25 °C_._ We observed that 39% of *csr-1b* mutant sperm are activated after one generation raised at 25 °C, in contrast with 97% activation rate in the control animals (Fig. 3c). This rate of spermatid activation drops further to 22% after two generations at 25 °C while the rate remains at 97% in the control animals. Furthermore, among the population of activated sperm in the *csr-1b* mutant, we occasionally observed a striking phenotype of spiky pseudopods, reminiscent of the compromised sperm of the *alg-3/4* mutants (Supplementary Fig. 3e)^33^. This phenotype was never observed in wild-type or *csr-1a* mutants. Thus, these data demonstrate that both CSR-1 isoforms are required for robust sperm activation.

Together, these data demonstrate that CSR-1A and CSR-1B do not depend on one another for localization or expression, and thus they behave as independent proteins with seemingly specialized functions in the *C. elegans* germline. More importantly, the data suggest that both CSR-1 isoforms are intimately involved in optimizing sperm health, though the mechanism used by each isoform to achieve this end still needs to be determined.

### CSR-1A and CSR-1B target distinct groups of genes

**Fig. 4:**
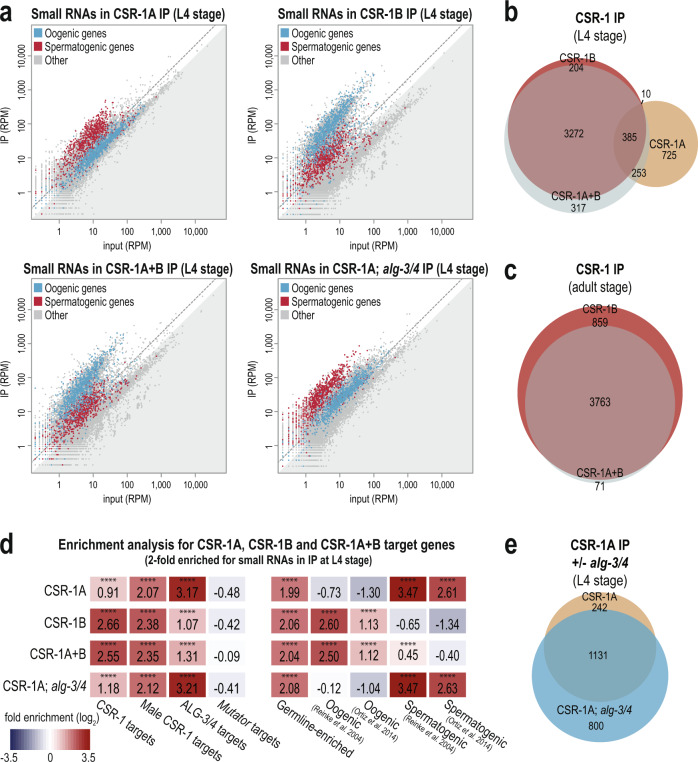
CSR-1A and CSR-1B target distinct groups of genes. **a** Normalized reads for oogenesis and spermatogenesis genes at L4 stage from HA::CSR-1A IP, FLAG::CSR-1B IP, FLAG::CSR-1A + B IP, and HA::CSR-1A; *alg-3/4* IP compared to input. Oogenic and spermatogenic genes (defined by Reinke et al., 2004) are indicated in blue and red, respectively. Gray dotted line indicates twofold enrichment in IP relative to input. **b** Venn diagram indicates overlap of CSR-1A, CSR-1B, and CSR-1A + B target genes at L4 stage. Target genes are defined are at least twofold enriched in the IP, with at least 10 RPM in IP samples and a DESeq2 adjusted *p* value ≤0.05. **c** Venn diagram indicates overlap of CSR-1B and CSR-1A + B gene lists at adult stage. **d** Enrichment analysis (log_2_(fold enrichment)) examining the overlap of CSR-1A, CSR-1B, and CSR-1A + B target genes with known targets of the CSR-1, male CSR-1, ALG-3/4, and *mutator* small RNA pathways and oogenesis and spermatogenesis-enriched genes. See Materials and Methods for gene list information. Two-tailed *p* values for enrichment was calculated using the Fisher’s exact test function in R. n.s. denotes not significant and indicates a *p* value >0.05 and **** indicates a *p* value ≤0.0001. See Supplementary Data 7 for more details regarding statistical analysis. **e** Venn diagram indicates overlap of CSR-1A target genes in the presence or absence of *alg-4*; *alg-3*.

To investigate the small RNAs bound by each CSR-1 isoform and thus determine whether CSR-1A and CSR-1B have distinct small RNA partners, we immunoprecipitated CSR-1A (from the *2xHA::csr-1a* strain), CSR-1B (from the *csr-1a*(*cmp135*); *2xFLAG::csr-1b* strain), and CSR-1A + B (from the *2xFLAG::csr-1b* strain), and sequenced the associated small RNAs. CSR-1A, CSR-1B, and CSR-1A + B were immunoprecipitated from L4 stage animals, and CSR-1B and CSR-1A + B were additionally immunoprecipitated from the adult stage, for comparison. We found that the small RNAs that immunoprecipitate with CSR-1A, CSR-1B, and CSR-1A + B are enriched for germline genes^34^ at both L4 and adult stages (Fig. 4a and Supplementary Fig. 4a). However, CSR-1A preferentially associates with small RNAs mapping to spermatogenic genes^34^ at the L4 stage while CSR-1B preferentially associates with small RNAs mapping to oogenic genes^34^ both at L4 and adult stages (Fig. 4a and Supplementary Fig. 4a). CSR-1B is expressed at much higher levels than CSR-1A at the L4 stage (Figs. 1b, 3f), therefore we would expect that when we tag both isoforms together (CSR-1A + B) the majority of the small RNAs immunoprecipitated would associate with CSR-1B. Indeed, we observed that the majority of the small RNAs immunoprecipitated with CSR-1A + B map to oogenic genes at both L4 and adult stages (Fig. 4a and Supplementary Fig. 4a). Regardless of isoform, the majority of the small RNAs associated with CSR-1 are 22-nucleotide long with a strong bias for guanine as the 5′ nucleotide (Supplementary Fig. 4b–d). These data indicate that, while both isoforms of CSR-1 bind 22G-RNAs mapping to germline genes, CSR-1A is enriched for siRNAs mapping to spermatogenesis-specific genes and CSR-1B is enriched for siRNAs mapping to oogenesis-specific genes.

We next sought to define a list of genes targeted by CSR-1A and CSR-1B. We defined the CSR-1A, CSR-1B, and CSR-1A + B target genes at both L4 and adult stages as those with complementary small RNAs at least twofold enriched in the IP compared to input, with at least 10 RPM in the IP samples and a DESeq2 adjusted *p* value of ≤0.05 (Supplementary Data 1). Comparing these gene lists with one another, we found that the majority of CSR-1B target genes are also CSR-1A + B target genes at both L4 (94.5% overlap) and adult stages (81.4% overlap) (Fig. 4b, c). Furthermore, the CSR-1B target genes at L4 stage overlap significantly with the CSR-1B target genes at adult stage (94.0% overlap), and similarly the CSR-1A + B target genes at L4 stage overlap significantly with the CSR-1A + B target genes at adult stage (78.0% overlap) (Supplementary Fig. 4e, f). In contrast, only 28.8% of CSR-1A target genes at L4 stage overlap with CSR-1B target genes at L4 stage, and only 46.6% of CSR-1A target genes at L4 stage overlap with CSR-1A + B target genes at L4 stage, despite the CSR-1A + B IP at L4 stage immunoprecipitating both isoforms (Fig. 4b). Thus, these data demonstrate that CSR-1A targets a distinct set of genes and that due to the much higher expression of CSR-1B, immunoprecipitation of the two isoforms together at the L4 stage enriches for small RNAs that predominantly map to CSR-1B target genes.

To further our understanding of which genes are targeted by CSR-1A and CSR-1B, we compared the CSR-1A, CSR-1B, and CSR-1A + B target gene lists to previously described target gene lists for other small RNA pathways^28,33–39^. As expected, CSR-1B and CSR-1A + B are strongly enriched for hermaphrodite and male CSR-1 target genes, and more modestly enriched for ALG-3/4 target genes (Fig. 4d). In contrast, CSR-1A is only modestly enriched for hermaphrodite CSR-1 target genes, and strongly enriched for ALG-3/4 target genes and male CSR-1 target genes (Fig. 4d). It has previously been shown that male CSR-1 target genes, identified by immunoprecipitating both isoforms of CSR-1 in males, include both oogenesis-specific genes identified as CSR-1 targets in hermaphrodites, and male-specific genes that are also ALG-3/4 targets^33^. Thus, it is not surprising that both CSR-1B, which preferentially binds hermaphrodite CSR-1 target genes, and CSR-1A, which preferentially binds ALG-3/4 target genes, are enriched for male CSR-1 target genes (Fig. 4d). In further agreement with these data, the CSR-1A target gene list is strongly enriched for spermatogenic genes, and the CSR-1B and CSR-1A + B target gene lists are strongly enriched for oogenic genes (Fig. 4d). In contrast, neither CSR-1A, CSR-1B, nor CSR-1A + B are enriched for *mutator* target genes, which include pseudogenes, repetitive elements, transposons, and other germline-repressed genes (Fig. 4d). Together, these data further show that CSR-1A and CSR-1B bind small RNAs targeting germline genes associated with distinct small RNA pathways, with CSR-1A preferentially targeting spermatogenic genes and ALG-3/4 targets and CSR-1B preferentially targeting oogenic genes and hermaphrodite CSR-1 targets.

### CSR-1A does not require ALG-3/4 to bind spermatogenic small RNAs

Because CSR-1A shares a similar expression pattern to ALG-3 and targets spermatogenic genes, we next sought to determine whether the small RNAs bound to CSR-1A require ALG-3/4 for their production. To this end, we immunoprecipitated CSR-1A in an *alg-3/4* mutant background and sequenced the associated small RNAs. Interestingly, in the absence of *alg-3* and *alg-4*, CSR-1A was still preferentially loaded with small RNAs targeting spermatogenic genes (Fig. 4a, d). In addition, 82.4% of CSR-1A target genes in the wild-type background were also defined as targets of CSR-1A in the *alg-3*; *alg-4* double mutant background (Fig. 4e). Furthermore, CSR-1A target genes in the *alg-3*; *alg-4* double mutant background were strongly enriched for ALG-3/4 target genes and male CSR-1 target genes, and modestly enriched for hermaphrodite CSR-1 target genes, similar to CSR-1A target genes in a wild-type background (Fig. 4d). Thus, these data show, despite CSR-1A and ALG-3/4 targeting many of the same genes, CSR-1A is not directly downstream of ALG-3/4 in regulating spermatogenic gene expression and ALG-3 and ALG-4 are dispensable for the production of CSR-1A-bound spermatogenic small RNAs.

### CSR-1A expression is positively correlated with the expression of CSR-1A target genes

**Fig. 5:**
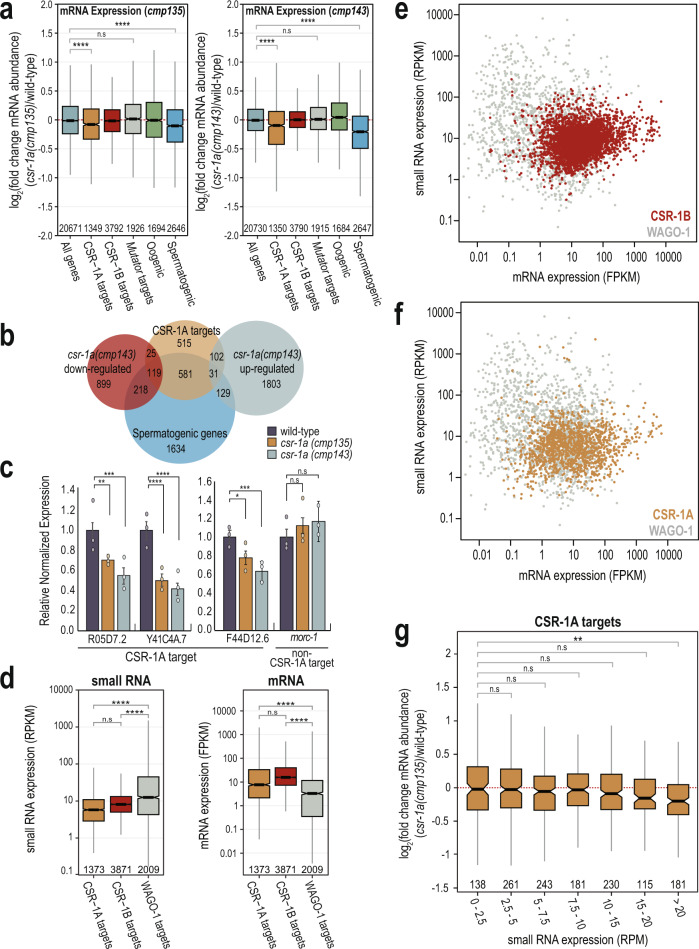
CSR-1A targets spermatogenesis-expressed genes. **a** Box plots depicting log_2_(fold change mRNA abundance) for L4 stage *csr-1a*(*cmp135*) (left) and *csr-1a*(*cmp143*) (right) mutants relative to wild-type animals at 20 °C (three biological replicates). Gene lists defined by Ortiz et al., 2014, Manage et al., 2020, and this work. **b** Venn diagram indicates overlap of CSR-1A targets, spermatogenic genes (Ortiz et al., 2014), and *csr-1a*(*cmp143*) up- and downregulated genes, which were defined as having a DESeq2 adjusted *p* value ≤0.05. **c** RT-qPCR from wild-type, *csr-1a* (*cmp135*), and *csr-1a* (*cmp143*) L4 animals raised at 20 °C for R05D7.2, Y41C4A.7, and F44D12.6, genes that are highly enriched in HA::CSR-1A IPs, and *morc-1*, which is enriched in FLAG::CSR-1B and FLAG::CSR-1A + B IPs, but not HA::CSR-1A IPs. Relative expression was normalized over *rpl-32* and calculated relative to wild-type animals. Bar graphs representing mean for three biological replicates and error bars indicate SEM. **d** Box plot depicting small RNA reads per kilobase per million (RPKM, left) and mRNA fragments per kilobase per million (FPKM, right) from L4 wild-type animals, raised at 20 °C, 48 h post L1 (two biological replicates for small RNA and three biological replicates for mRNA). Small RNAs and mRNAs transcripts were grouped based on their mapping to CSR-1B, CSR-1A, and WAGO-1 target genes, identified from the RNA-IP experiments. **e**, **f** Small RNA expression (RPKM) plotted against mRNA expression (FPKM) in wild-type animals for CSR-1B (**e**) and CSR-1A (**f**) target genes compared against WAGO-1 target genes. CSR-1B, CSR-1A, and WAGO-1 gene lists come from FLAG::CSR-1B IP, HA::CSR-1A IP, and FLAG::WAGO-1 IP in L4 animals, respectively. Each point is calculated from the average of two biological replicates for small RNA and three biological replicates for mRNA. **g** Box plot depicting log_2_(fold change mRNA abundance) of CSR-1A target genes for L4 stage *csr-1a*(*cmp135*) mutants relative to wild-type animals at 20 °C where the CSR-1A target genes are binned based on the mapped small RNA reads per million (RPM) in wild-type animals (three biological replicates). For all box plots (**a**, **d**, **g**), bolded midline indicates median value, box indicates the first and third quartiles, and whiskers represent the most extreme data points within 1.5 times the interquartile range, excluding outliers. Two-tailed *t*-tests were performed to determine statistical significance (**a**, **c**, **d**, **g**) and *p* values were adjusted for multiple comparisons (**a**, **d**, **g**). n.s denotes not significant and indicates a *p* value >0.05, * indicates a *p* value ≤0.05, ** indicates a *p* value ≤0.01, *** indicates a *p* value ≤0.001, and **** indicates a *p* value ≤0.0001. See Supplementary Data 7 for more details regarding statistical analysis.

It is well-established that CSR-1 targets germline-expressed genes, and that CSR-1 can either license and protect these transcripts from silencing by the piRNA pathway in the adult germline or can modestly tune the transcript level in developing oocytes and embryos^8,14,20^. To determine whether CSR-1A is similarly protecting or tuning its target transcripts, we generated mRNA high-throughput sequencing libraries from wild-type, *csr-1a*(*cmp135*) mutants, and *csr-1a*(*cmp143*) mutants at 20 °C (Supplementary Data 2). In both *csr-1a* mutant alleles, CSR-1A targets are modestly, but significantly, downregulated compared to wild-type (Fig. 5a). Similarly, spermatogenic genes^28^ are significantly downregulated in both *csr-1a* mutants compared to wild-type (Fig. 5a). In contrast, *mutator* target genes^40^ were unchanged in both *csr-1a* mutants (Fig. 5a). It is worth noting, however, that only 10.5% of CSR-1A targets are significantly downregulated (DESeq2 adjusted *p* value of ≤0.05) in *csr-1a*(*cmp143*) mutants, while 9.7% of CSR-1A targets are significantly upregulated (DESeq2 adjusted *p* value of ≤0.05) in *csr-1a*(*cmp143*) mutants (Fig. 5b). Of the downregulated CSR-1A targets, 82.6% are also spermatogenic genes^28^, while the upregulated CSR-1A targets only include 23.3% spermatogenic genes (Fig. 5b). To confirm that some CSR-1A target transcripts are indeed reduced in the *csr-1a* mutants, we performed RT-qPCR on three spermatogenic genes that were strongly enriched for mapped small RNAs in the CSR-1A immunoprecipitation. We determined that all three genes showed a consistent and significant decrease in transcript levels in both *csr-1a* mutants compared to wild-type animals (Fig. 5c). In contrast, the *csr-1a* mutants had no effect on *morc-1*, a germline gene that was only enriched in CSR-1B and CSR1A + B immunoprecipitation, and not in CSR-1A (Fig. 5c). Therefore, CSR-1A appears to have modest but significant positive effects on some of its target transcripts, such that in its absence, these genes have reduced expression.

Recent studies have examined the relationship between the levels of siRNAs targeting each transcript in correlation with mRNA expression^20,41^. In general, mRNAs expression tends to be lower for genes with very high levels of siRNAs and for genes that are targets of the Argonaute protein, WAGO-1^41^. In contrast, mRNA expression tends to be higher for genes that are targets of CSR-1^41^. To determine whether this trend holds true for CSR-1A target genes, for comparison, we generated a list of WAGO-1 target genes at the L4 stage by immunoprecipitating WAGO-1 and defining targets as those at least twofold enriched in a WAGO-1 IP compared to input, with at least 10 RPM in the IP samples, and a DESeq2 adjusted *p* value of ≤0.05. Then using mRNA and small RNA sequencing libraries from wild-type animals at L4 stage, we found that WAGO-1 targets have significantly higher levels of mapping complementary small RNAs compared to CSR-1A and CSR-1B targets (Fig. 5d–f). Moreover, mRNA expression of WAGO-1 targets is significantly lower than mRNA expression of CSR-1A and CSR-1B target genes (Fig. 5d–f). Therefore, the CSR-1A bound small RNAs and target mRNAs are more similar in expression level to those of CSR-1B compared to WAGO-1, further supporting the argument that CSR-1A may function similarly to CSR-1B to promote expression of its target genes. We next grouped the CSR-1A target genes into seven bins based on their small RNA expression in wild-type animals. We found that higher levels of CSR-1A siRNAs (>20 RPM in wild-type animals) correlates with reduced mRNA expression in *csr-1a* mutants, while the expression of genes with lower levels of CSR-1A siRNAs tends to be unchanged in *csr-1a* mutants (Fig. 5g). This result is consistent with both the very modest effect of the *csr-1a* mutants on transcript level of all CSR-1A target genes (Fig. 5a) and the much more significant effect of the *csr-1a* mutants on the transcript level of the three CSR-1A target genes tested by qPCR (Fig. 5c), which were selected based on the strong enrichment for their mapped small RNAs in the CSR-1A immunoprecipitation. Together, these data suggest that CSR-1A may share a similar licensing mechanism to what has been previously proposed for CSR-1, and that in *csr-1a* mutants, the spermatogenic transcripts licensed by CSR-1A are no longer protected and may be subjected to degradation.

### The first exon of CSR-1A is unstructured and contains RG motifs

CSR-1A differs from its shorter counterpart by a single N-terminal exon. The exon is arginine/glycine-rich (containing RG motifs), and we sought to determine whether this unique exon is conserved across the *Caenorhabditis* genus. We first identified CSR-1 orthologs in several closely related nematode species, including *C. brenneri*, *C. briggsae*, *C. japonica*, *C. latens*, *C. nigoni*, *C. remanei*, *C. sinica*, and *C. tropicalis*. The first exon of CSR-1 in each species is the least conserved portion of the protein; however, like *C. elegans* CSR-1A, each ortholog had enrichment of arginines and glycines in this region (Supplementary Fig. 5). Furthermore, while only a single isoform is annotated in most of the species, all orthologs possessed a conserved methionine at the position of the *C. elegans* CSR-1B start codon (Supplementary Fig. 5). Additionally, all eight orthologs have a large intron between the first exon and the rest of the protein, with a median size of 617 bp and the smallest being 503 bp in *C. tropicalis*. These data suggest that the regulatory elements driving CSR-1B expression in *C. elegans*, which are found in this intron, may also be conserved.

Because of the repetitive nature of the RG motif region and the lack of otherwise strong sequence conservation, we next asked whether the first exon of CSR-1 contains regions of intrinsic disorder. Using IUPred2^42,43^, we determined that the first exon of CSR-1A in *C. elegans* is highly disordered while the rest of the protein is predicted to be structured (Supplementary Fig. 6a). We then examined the CSR-1 orthologs in related *Caenorhabditis* species and found that, like in *C. elegans*, the first exon of CSR-1 is highly disordered in all related nematode species (Supplementary Fig. 6b), demonstrating that the disordered nature of the first exon is a conserved feature of the CSR-1 protein.

### The RG motifs in the first exon of CSR-1A are dimethylated

**Fig. 6:**
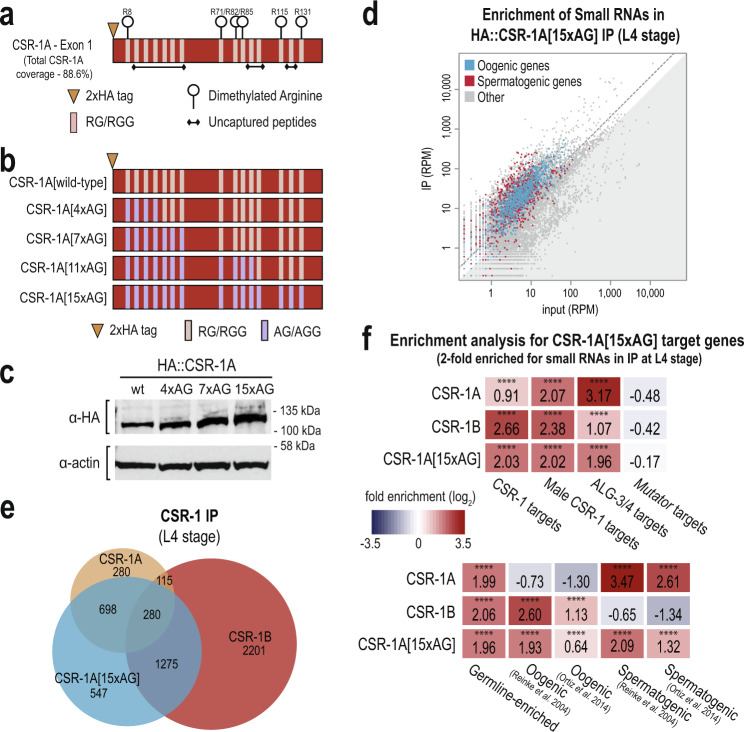
Methylation of CSR-1A N-terminal exon promotes binding preference for spermatogenic siRNAs. **a** Graphical display of dimethylation modifications detected on CSR-1A by mass spectrometry following IP. **b** Schematic representation of the series of arginine to alanine mutations created by CRISPR to generate the unmethylatable CSR-1A[15xAG]. **c** Western blot for HA::CSR-1A in the RG-to-AG mutants. Actin is shown as a loading control. The blot has been reproduced. **d** Normalized reads for spermatogenic and oogenic genes (defined by Reinke et al., 2004) from HA::CSR-1A[15xAG] IP compared to input. Spermatogenic and oogenic genes are highlighted in red and blue, respectively. Gray dotted line indicates twofold enrichment in IP relative to input. **e** Venn diagram indicates overlap of CSR-1A, CSR-1B, and CSR-1A[15xAG] target genes at L4 stage. **f** Enrichment analysis (log_2_(fold enrichment)) examining the overlap of CSR-1A, CSR-1B, and CSR-1A[15xAG] target genes with known targets of the CSR-1, male CSR-1, ALG-3/4, and *mutator* small RNA pathways and oogenesis and spermatogenesis-enriched genes. See Materials and Methods for gene list information. Two-tailed *p* values for enrichment was calculated using the Fisher’s exact test function in R. n.s denotes not significant and indicates a *p* value >0.05 and **** indicates a *p* value ≤0.0001. See Supplementary Data 7 for more details regarding statistical analysis. Source data are provided as a Source data file.

It has been shown previously that RG motifs are often targets of Protein arginine methyltransferases (PRMTs), which catalyze the methylation of arginine residues^44–47^. To determine whether CSR-1A contains methylated arginines, we immunoprecipitated 2xHA::CSR-1A and subjected the sample to mass spectrometry analysis. We identified six dimethylated arginines in the first exon of CSR-1A, which constituted 100% of the RG motifs captured by mass spectrometry (Fig. 6a and Supplementary Data 3). The remaining RG motifs were not captured, methylated or unmethylated, by our mass spectrometry experiment. We did not identify any dimethylarginines in the portion of the CSR-1 protein found in both CSR-1A and CSR-1B isoforms, however the conserved RGRG sequence found near the N-terminus of CSR-1B, a good candidate region for dimethylation, was also not captured by our mass spectrometry experiment. We were also unable to determine whether these arginines were symmetrically or asymmetrically dimethylated, which will be an important distinction for future studies. Together, these data indicate that the first exon of CSR-1A is heavily methylated, and, given the conservation of RG motifs in this region across *Caenorhabditis* species, this methylation is likely also conserved.

We next sought to study the function of the dimethylarginine modification. To this end, we mutated all arginines found in RG motifs within the first exon of CSR-1A to alanine using CRISPR, thus rendering the exon unmethylatable. Because we generated the mutations sequentially from the N-terminus of the protein, three-to-four arginines at a time, we created a series of proteins with four, seven, 11 or all 15 RG motifs mutated to AG— hereafter referred to as CSR-1A[4xAG], CSR-1A[7xAG], CSR-1A[11xAG], and CSR-1A[15xAG] (Fig. 6b). We first asked if dimethylation of arginines affects the expression or stability of the CSR-1A protein. By western blot, we determined that the CSR-1A[4xAG], CSR-1A[7xAG], and CSR-1A[15xAG] proteins are expressed at levels comparable to wild-type (Fig. 6c). Because dimethylation has been shown previously to contribute to Argonaute protein localization^48^, we next tested whether dimethylation is necessary for CSR-1A localization to P granules. We performed immunostaining on CSR-1A[wild-type], CSR-1A[4xAG], and CSR-1A[15xAG] L4 stage hermaphrodites and detected no discernable differences in localization in the RG-to-AG mutant strains (Supplementary Fig. 6c). Finally, to determine whether the dimethylated RG motifs are required for fertility, we performed brood size assays on CSR-1A[wild-type], CSR-1A[7xAG], and CSR-1A[15xAG] animals. We found that CSR-1A[7xAG] and CSR-1A[15xAG] animals have a modest but significant reduction in brood size compared to the control strain, indicating that *csr-1a* RG motif mutants phenocopy a null *csr-1a* mutant (Supplementary Fig. 6d). Therefore, our data demonstrates that the first exon of CSR-1A contains the dimethylarginine modification, however this modification is not required for expression or localization of the CSR-1A protein. Furthermore, since the brood sizes were similar between CSR-1A[7xAG] and CSR-1A[15xAG], this data may suggest that the seven most N-terminal RG sites are more crucial or that a minimum number of methylated sites is required for optimal CSR-1A function.

### The RG motifs in CSR-1A promote specificity for binding small RNAs targeting spermatogenic genes

CSR-1A and CSR-1B both localize to P granules in the spermatogenesis region of the germline in L4 stage hermaphrodites and males, yet each isoform has distinct small RNA targets (Figs. 1a and 4a, b). Because the isoforms only differ by the presence of the RG motif-containing exon in CSR-1A, we asked if the RG motifs and the dimethylarginine modification could be a mechanism by which CSR-1A preferentially associates with spermatogenic small RNAs. To test this hypothesis, we immunoprecipitated 2xHA::CSR-1A[15xAG] from L4 stage animals and subjected the associated small RNAs to high-throughput sequencing. We found that the small RNAs that immunoprecipitate with CSR-1A[15xAG] map to both spermatogenic and oogenic genes, with no discernable preference for either (Fig. 6d). We next defined the list of genes targeted by CSR-1A[15xAG] as those at least twofold enriched in the IP compared to input, with at least 10 RPM in the IP samples and a DESeq2 adjusted *p* value of ≤0.05 (Supplementary Data 1). Comparing this gene list to the wild-type CSR-1A and CSR-1B target gene lists at L4 stage, we found that 34.9% (978 genes) of the genes in the CSR-1A[15xAG] list overlapped with the wild-type CSR-1A gene list and 55.5% (1555 genes) of the genes in the CSR-1A[15xAG] list overlapped with the CSR-1B gene list (Fig. 6e). Furthermore, when we compared the CSR-1A[15xAG] target gene list to previously described target genes lists for other small RNA pathways^28,33–39^, we found that the CSR-1A[15xAG] target gene list is strongly enriched for hermaphrodite and male CSR-1 target genes, and for ALG-3/4 target genes (Fig. 6f). Additionally, the CSR-1A[15xAG] target gene list is strongly enriched for both spermatogenic and oogenic genes (Fig. 6f). These results are in contrast to the wild-type CSR-1A target gene list, which is strongly enriched for spermatogenic genes but not oogenic genes, and to the CSR-1B target gene list, which is strongly enriched for oogenic genes but not spermatogenic genes (Fig. 6f). However, like wild-type CSR-1A and CSR-1B, CSR-1A[15xAG] is not enriched for *mutator* target genes (Fig. 6f). Together, these data indicate that loss of the RG motifs, and therefore loss of the dimethylarginine modification, is associated with a reduced specificity of CSR-1A for small RNAs targeting spermatogenesis genes and an increased binding of oogenic small RNAs.

## Discussion

The Argonaute protein CSR-1 is critical to promote germline gene expression and fertility. Here we demonstrate that, in addition to the short isoform CSR-1B which is expressed throughout germline development and during gametogenesis of both sexes, a longer isoform, CSR-1A, is selectively expressed during spermatogenesis. During L4, where both isoforms are expressed in the spermatogenesis region, CSR-1A and CSR-1B co-localize at P granules. Yet despite localizing to the same subcellular structure and sharing nearly complete sequence and presumably structural identity, CSR-1A and CSR-1B associate with distinct subsets of small RNAs targeting germline-expressed genes—CSR-1A preferentially binds to small RNAs targeting spermatogenic genes, whereas CSR-1B binds to small RNAs targeting oogenic genes. The single exon unique to CSR-1A contains RG motifs that are modified with dimethylarginine and these methylated RG motifs are critical to CSR-1A specificity for spermatogenic small RNA substrates.

### CSR-1A promotes spermatogenic gene expression

Previous work has shown that CSR-1 can license targeted transcripts for expression in the adult germline, while its catalytic domain is necessary to tune embryonic gene expression and clear maternal transcripts^13,14,20,49^. Though these studies did not make a distinction between CSR-1A and CSR-1B, based on the expression of only CSR-1B in the adult germline and embryos, we can attribute these CSR-1 activities to the CSR-1B isoform. Here we show that CSR-1A similarly does not downregulate its target genes during spermatogenesis and in fact, seems to modestly promote the expression of some spermatogenic genes. RNAi of CSR-1 has also been demonstrated to trigger mis-expression of spermatogenic mRNAs in the germlines of adult-stage animals, which, under normal conditions, should be undergoing oogenesis^50^. This study also did not distinguish between CSR-1A and CSR-1B isoforms, therefore it remains to be determined which CSR-1 isoform is required for suppressing spermatogenic transcription during oogenesis.

The Argonaute proteins ALG-3 and ALG-4 similarly promote the expression of many spermatogenic genes, and trigger the biogenesis of ALG-3/4-dependent 22G-RNAs as part of a feed-forward loop to promote paternal inheritance^26,33^. With ALG-3 and CSR-1A sharing nearly identical expression patterns and CSR-1A being a spermatogenic Argonaute with a preference for spermatogenic 22G-RNAs, an obvious hypothesis would be that CSR-1A acts downstream of ALG-3 and ALG-4. However, the CSR-1A spermatogenic 22G-RNAs are produced independently of ALG-3/4 indicating that CSR-1A is likely part of a distinct pathway that targets the same spermatogenic genes as ALG-3/4 and another still-unidentified Argonaute binds the ALG-3/4-dependent 22G-RNAs. Importantly, these findings do not exclude the possibility of CSR-1B being an essential player in paternal epigenetic inheritance and acting downstream of ALG-3/4, as CSR-1B is still present in secondary spermatocytes with ALG-3^33^, when CSR-1A has already disappeared. At the L4 stage, when both isoforms are expressed, our immunoprecipitation and sequencing of CSR-1B-bound small RNAs indicate that CSR-1B targets gender-neutral and oogenic mRNAs. It is important to note that, during the L4 stage, CSR-1B is expressed throughout the germline while CSR-1A and ALG-3 are restricted to the spermatogenic region (Fig. 2b and Supplementary Fig. S2a)^26^. Thus, when sequencing small RNAs immunoprecipitated from whole animals, we are unable to determine whether CSR-1B is binding oogenic siRNAs throughout the germline or in specific germline regions, and whether CSR-1B could bind low levels of spermatogenic siRNAs during spermatogenesis that fall below our cutoff for enrichment. Furthermore, since the majority of CSR-1 studies have examined adults or embryos^8,13,14,20^, it is unclear whether, during the L4 stage, CSR-1B is licensing or repressing its target mRNAs, or even protecting them from ALG-3/4 or CSR-1A targeting. Or perhaps oogenic mRNAs are at such low levels throughout development that most CSR-1B Argonaute proteins remain unassociated with mRNA until the transition to adulthood and the onset of oogenesis.

If CSR-1A does not act downstream of ALG-3/4 for paternal inheritance, then how might it function to promote spermatogenic gene expression? If we look to CSR-1B as a guide, then perhaps CSR-1A acts to license spermatogenic transcripts for expression only in the L4 and male germlines by protecting them from targeting by piRNAs and the WAGO clade of Argonaute proteins. CSR-1A could further tune spermatogenic gene expression or clear spermatogenic transcripts at the spermatogenesis to oogenesis transition. Alternatively, the role for CSR-1A may be to sequester the abundant sperm transcripts and spermatogenic small RNAs away from CSR-1B, so that the dominant isoform can appropriately bind to its oogenic small RNA targets. Through this lens, CSR-1A serves almost exclusively to titrate CSR-1B siRNA levels targeting oogenic genes and might explain the modest effect on the sperm transcripts upon removing CSR-1A. Further experiments will be needed to sort out these possibilities.

### Dimethylarginine promotes isoform-specific small RNA loading

Posttranslational modification has been shown to play a key role for Argonaute function in *C. elegans* and in other systems. The *C. elegans* miRNA Argonaute protein, ALG-1, contains a cluster of phosphorylation sites that is conserved in human Argonaute Ago2, demonstrating that these modification sites are highly conserved between species. The function of this phosphorylation also appears to be conserved—the phosphorylated Argonaute proteins cannot associate with mRNAs, suggesting the role of the modification may be in mediating release of mRNA-Argonaute complexes for recycling^51^. PIWI proteins from mouse, *Xenopus laevis*, and *Drosophila melanogaster* contain symmetrical dimethylarginines (sDMA), and in *Drosophila* this modification is mediated by protein arginine methyltransferase PRMT5^44,46,52^. The dimethylarginine modification allows the Argonaute protein to interact with members of the Tudor domain protein family^46–48,52,53^. Tudor domains, which are protein–protein interaction modules, recognize methylated arginines and thus can mediate protein–protein interactions in a methylation-specific manner. Furthermore, for some PIWI proteins, subcellular localization to nuage is dependent on its interaction with Tudor proteins^46,48,52^.

Here, we have found the first exon of CSR-1A to be highly modified with dimethylarginine. Interestingly, dimethylarginine is not required for CSR-1A localization to the P granule, but instead for small RNA specificity of CSR-1A, and thus recognition of the correct target transcripts. We hypothesize that CSR-1A interacts with an unknown Tudor domain protein through its dimethylated RG motifs, to promote engagement with a distinct small RNA biogenesis complex. In this scenario, dimethylarginine provides a new binding platform for proteins that cannot associate with CSR-1B, allowing CSR-1A to make distinct protein–protein interactions and ultimately to target a unique subset of genes. It is interesting to note that the majority of the RG motifs, including all methylated sites captured in our mass spectrometry experiment, are found in the exon unique to CSR-1A and absent from CSR-1B, making this highly modified region isoform-specific. There are several other *C. elegans* Argonaute proteins with multiple splice variants, including the miRNA Argonaute proteins ALG-1 and ALG-2, the oogenesis-specific primary Argonaute protein ERGO-1, and the WAGO-clade Argonaute protein PPW-1. It is currently unknown whether the isoforms of any of these genes have distinct functions or unique protein modifications, or if CSR-1 is unique in this regard.

## Methods

### *C. elegans* strains

Strains were maintained at 20 °C on NGM plates seeded with OP50 *E. coli* according to standard conditions unless otherwise stated^54^. All strains used in this project are listed in Supplementary Data 4.

### Plasmid and strain construction

#### Plasmid-based CRISPR

All fluorescent and epitope tags were integrated at the endogenous loci by CRISPR genome editing^55–59^. For all CRISPR insertions of fluorescent tags, we generated homologous repair templates using the primers listed in Supplementary Data 5. Design of the *2xHA::mCherry* plasmid was described previously^60^. The *2xHA::mCherry*[*w/internal Floxed Cbr-unc-119*(*+*)] was amplified by PCR and assembled by isothermal cloning with ~1.5 kb of sequence from either side of the *csr-1a* start codon^61^. *gfp::3xFLAG::wago-1*, *gfp::3xFLAG::csr-1a* + *b* and *gfp::3xFLAG::alg-3* were assembled into pDD282 (Addgene #66823) by isothermal assembly according to published protocols^57,61^. To protect the repair template from cleavage, we introduced silent mutations at the site of guide RNA targeting by incorporating these mutations into one of the homology arm primers or, if necessary, by performing site-directed mutagenesis^56^. All guide RNA plasmids were generated by ligating oligos containing the guide RNA sequence into BsaI-digested pRB1017 (Addgene #59936)^59^. Guide RNA sequences are provided in Supplementary Data 5. GFP/mcherry CRISPR injection mixes included 25–50 ng/μl repair template, 50 ng/μl guide RNA plasmid, 50 ng/μl *eft-3p::cas9-SV40_NLS::tbb-2 3*′*UTR* (Addgene #46168), 2.5–10 ng/μl GFP or mCherry co-injection markers. The *2xHA::mCherry::csr-1a* construct was injected into USC868 (*mut-16(cmp3[mut-16::gfp* + *loxP*]) *I*; *unc-119*(*ed3*) *III*), the *gfp::3xFLAG::wago-1* construct was injected into USC896 (*mut-16(cmp41*[*mut-16::mCherry::2xHA* + *loxP*]) *I*), the *gfp::3xFLAG::csr-1a* + *b* construct was injected into the wild-type strain, and the *gfp::3xFLAG::alg-3* construct was injected into both the wild-type strain and USC1066 (*csr-1a(cmp90*[(*2xHA* + *mCherry* + *loxP Cbr-unc-119*(*+*) *loxP*)*::csr-1a*]) *IV*)^56,57^. For *csr-1a* deletions (*cmp135* and *cmp143*), *2xHA::csr-1a*, and *2xFLAG::csr-1a* + *b*, the injection mixes included 50 ng/μl repair oligo, 25 ng/μl guide RNA plasmid, 50 ng/μl pha-1 repair template, and *eft-3p::Cas9* + *pha-1* guide (pJW1285, Addgene #61252). GE24 (*pha-1(e2123*) *III*) mutant animals were injected and subsequently shifted to restrictive temperature (25 °C). Surviving F1 progeny were genotyped by PCR to identify the deletions and insertions of interest^58^. For *2xFLAG::csr-1b*, the injection mix included 50 ng/μl *csr-1b* repair oligo, 25 ng/μl *csr-1b* guide RNA plasmid, 20 ng/μl *dpy-10* repair template, 25 ng/μl *dpy-10* guide RNA (pJA58, Addgene #59933), and 50 ng/μl *eft-3p::Cas9* (pJW1259, Addgene #61251). Mixture was injected into USC1065 (*csr-1a(cmp135*) *IV*) mutant animals. F1 animals with the Rol phenotype were isolated and genotyped by PCR to identify animals with the 2xFLAG insertion^59^. The *csr-1a* RG-to-AG mutants were created sequentially, starting with *csr-1a*[*7xAG*]. *csr-1a*[*4xAG*] was an incomplete repair event identified from the *csr-1a*[*7xAG*] injections. *csr-1a*[*11xAG*] was injected into the *csr-1a*[*7xAG*] mutant and *csr-1a*[*15xAG*] was subsequently injected into the *csr-1a*[*11xAG*] mutant. The *csr-1a RGG* injection mixes include 50 ng/μl repair template, 25 ng/μl each of two guide RNA plasmids, 25 ng/μl rol-6 guide RNA (pJA42, Addgene #59930), 20 ng/μl rol-6 repair template, and 50 ng/μl *eft-3p::Cas9* (pJW1259, Addgene #61251). F1 animals with the Rol phenotype were isolated and genotyped by PCR to identify animals with the RG-to-AG mutations^59^. All repair template sequences are provided in Supplementary Data 5.

#### Protein-based CRISPR

For *csr-1a* deletions (*cmp253* and *cmp254*), *csr-1b*(*cmp258*), and *2xHA::csr-1a* in the *alg-3*; *alg-4* mutant, we used an oligo repair template and RNA guide (Supplementary Data 5). The injection mixes for *csr-1b*(*cmp258*) and *2xHA::csr-1a* included 0.25 μg/μl Cas9 protein (IDT), 100 ng/μl tracrRNA (IDT), 14 ng/μl *dpy-10* crRNA, 42 ng/μl gene-specific crRNA, and 110 ng/μl of each repair template, and were injected into USC1066 (*csr-1a(cmp90*[(*2xHA* + *mCherry* + *loxP Cbr-unc-119*(*+*) *loxP*)*::csr-1a*]) *IV*) and WM200 (*alg-4*(*ok1041*) *III*; *alg-3*(*tm1155*) *IV*), respectively. The injection mix for *csr-1a*(*cmp253*) and *csr-1a*(*cmp254*) included 0.25 μg/μl Cas9 protein (IDT), 100 ng/μl tracrRNA (IDT), 14 ng/μl *dpy-10* crRNA, 21 ng/μl each gene-specific crRNA, and 110 ng/μl of each repair template, and was injected into the wild-type strain. The repair template was designed to create the *cmp254* mutation; the larger *cmp253* deletion was an incorrect repair event. Following injection, F1 animals with the Rol phenotype were isolated and genotyped by PCR to identify animals with the insertions and deletions of interest^62,63^.

#### MosSCI

*csr-1* mCherry and GFP promoter fusions were integrated by Mos-mediated single-copy transgene insertion (MosSCI)^29^. For the MosSCI insertions of promoter-fused GFP and mCherry, we amplified *csr-1a* and *csr-1b* endogenous promoters, the mCherry and GFP genes, and the *csr-1* 3′UTR using the primers listed in Supplementary Data 5. Plasmids were assembled by isothermal cloning^61^. For MosSCI injections, we integrated transgenes into the *ttTi5605 MosI* site in strain EG4322 (Ch. II) following a published MosSCI protocol^29^. Injection mixes contained 50 ng/μl MosSCI-targeting vector, 50 ng/μl *eft-3p*::*Mos1 transposase* (pCFJ601, Addgene #34874), 10 ng/μl *rab-3p*::*mCherry* (pGH8, Addgene #19359), 2.5 ng/μl *myo-2p*::*mCherry* (pCFJ90, Addgene #19327), 5 ng/μl *myo-3p*::*mCherry* (pCFJ104, Addgene #19328), and 10 ng/μl *hsp-16*.*1*::*peel-1*negative selection (pMA122, Addgene #34873)^29^.

### Antibody staining and imaging

Live imaging of *C*. *elegans* was performed in M9 buffer containing sodium azide to prevent movement. For immunofluorescence, *C. elegans* were dissected in egg buffer containing 0.1% Tween-20 and fixed in 1% formaldehyde in egg buffer as described^64^. Samples were immunostained with mouse anti-FLAG 1:500 (Sigma Aldrich, F1804), mouse anti-PGL-1 1:100 (DSHB K76)^65^, and rat anti-HA 1:500 (Roche, 11867423001). Alexa-Fluor secondary antibodies were purchased from Thermo Fisher. Animals were dissected at the L4 (48 h post hatching) or young adult stage (52 h post hatching). Imaging was performed on a DeltaVision Elite microscope (GE Healthcare) using a 60x N.A. 1.42 oil-immersion objective. When data stacks were collected, three-dimensional images are presented as maximum intensity projections. Images were pseudocolored using the SoftWoRx package or Adobe Photoshop.

### Western blots

*C. elegans* were synchronized at 20 °C by bleaching gravid adult animals and maintaining starved L1 larvae for at least 24 h before plating on OP50. For sample collection, animals were harvested after 2 h (L1), 12 h (L2), 32 h (L3), 50 h (L4), and 72 h (gravid adult) on OP50. Number of animals loaded per lane was normalized for actin—~1000 L1s, 800 L2s, 600 L3s, 400 L4s, and 200 gravid adults. Proteins were resolved on 4–12% Bis-Tris polyacrylamide gels (Thermo Fisher), transferred to nitrocellulose membranes (Thermo Fisher), and probed with rat anti-HA-peroxidase 1:1000 (Roche 12013819001), mouse anti-FLAG 1:1000 (Sigma, F1804), mouse anti-actin 1:10,000 (Abcam ab3280), or rabbit anti-CSR-1 1:2000 antibodies^8^. Secondary HRP antibodies were purchased from Thermo Fisher. Unedited western blots provided in the Source Data File.

### RNA isolation and qRT-PCR

Synchronized L4 stage animals (~48 h at 20 °C after L1 arrest) were collected. RNA was isolated using Trizol reagent (Thermo Fisher), followed by chloroform extraction and isopropanol precipitation. RNA samples were normalized to 10 μg/μL prior to DNase treatment (TURBO DNA-free kit, Thermo Fisher AM1907) and reverse transcribed with SuperScript III Reverse Transcriptase (Thermo Fisher 18080-051). All Real-time PCR reactions were performed using the 2x iTaq Universal SYBR Green Supermix (Bio-Rad 1725121), following the manufacturer’s protocols, and run in the CFX96 Touch Real-Time PCR System (Bio-Rad 1855195). Samples were run with three technical replicates and three biological replicates, and normalized to *rpl-32*. Primer sequences are available in Supplementary Data 5.

### Brood size analysis

Wild-type and mutant *C. elegans* strains were maintained at 20 **°**C prior to temperature-shift experiments. Animals were either maintained at 20 **°**C or shifted to 25 **°**C as L4 larvae and ten of their progeny were picked to individual plates for 25 °C brood size analysis. To score the complete brood, each animal was moved to a fresh plate every day until egg-laying was complete. After allowing the progeny 2–3 days to develop, the total number of animals on each plate was counted.

### In vitro sperm activation assay

Virgin L4 males were isolated 24 h before assay. About 10–15 males were dissected in 30 uL of 500 μg/mL Pronase E (Millipore Sigma), which was dissolved in sperm medium (50 mM HEPES, 50 mM NaCl, 25 mM KCl, 5 mM CaCl_2_, 1 mM MgSO_4_, 1 mg/ml BSA), as described previously^32,66^. Spermatids were incubated for 15 min at room temperature in a humid chamber before mounting and imaging on a DeltaVision Elite microscope (GE Healthcare) using a 60x N.A. 1.42 oil-immersion objective.

### Immunoprecipitations and mass spectrometry

For immunoprecipitation experiments followed by small RNA library preparation, ~100,000 synchronized L4 animals (~49 h at 20 °C after L1 arrest) or adult animals ~68 h at 20 °C after L1 arrest) were collected in IP Buffer (50 mM Tris-Cl pH 7.4, 100 mM KCl, 2.5 mM MgCl_2_, 0.1% Igapal CA-630, 0.5 mM PMSF, cOmplete Protease Inhibitor Cocktail (Roche 04693159001), and RNaseOUT Ribonuclease Inhibitor (Thermo Fisher 10777019)), frozen in liquid nitrogen, and homogenized using a mortar and pestle. After further dilution into IP buffer (1:10 packed worms:buffer), insoluble particulate was removed by centrifugation and 10% of sample was taken as “input.” The remaining lysate was used for the immunoprecipitation. Immunoprecipitation was performed at 4 °C for 1 h with pre-conjugated anti-HA affinity matrix (Roche 11815016001) or anti-FLAG affinity matrix (Sigma Aldrich A22220), then washed at least three times in immunoprecipitation buffer. A fraction of each sample was analyzed by western blot to confirm efficacy of immunoprecipitation. Trizol reagent (Thermo Fisher) was added to the remainder of each sample, followed by chloroform extraction, isopropanol precipitation, and small RNA library preparation.

For mass spectrometry experiments to identify posttranslational modifications, immunoprecipitation was performed as described above, starting with ~1.25 million synchronized USC1110 (*csr-1a*(*cmp165*[*2xHA::csr-1a*])) L4 stage animals (~48 h at 20 °C after L1 arrest). Wild-type animals were prepped alongside as a negative control. Immunoprecipitation was performed using anti-HA affinity matrix (Roche 11815016001). After immunoprecipitation, a fraction of each sample was analyzed by western blot to confirm efficacy of immunoprecipitation. 2x sample buffer was added to the remainder of each sample, followed by gel electrophoresis (4–12% Bis-Tris polyacrylamide gels, Thermo Fisher) and overnight colloidal Coomassie staining.

Bands containing immunoprecipitated protein were excised from gel and cut into ~1 mm^3^ pieces. Gel pieces were then subjected to a modified in-gel chymotrypsin digestion procedure^67^. Gel pieces were washed and dehydrated with acetonitrile for 10 min. followed by removal of acetonitrile. Pieces were then completely dried in a speed-vac. Rehydration of the gel pieces was with 50 mM ammonium bicarbonate solution containing 12.5 ng/µl modified sequencing-grade chymotrypsin (Promega) at 4 °C. After 45 min, the excess chymotrypsin solution was removed and replaced with 50 mM ammonium bicarbonate solution to just cover the gel pieces. Samples were then incubated at room temperature overnight. Peptides were later extracted by removing the ammonium bicarbonate solution, followed by one wash with a solution containing 50% acetonitrile and 1% formic acid. The extracts were dried in a speed-vac (~1 h) and stored at 4 °C until analysis.

On the day of analysis, the samples were reconstituted in 5–10 µl of HPLC solvent A (2.5% acetonitrile, 0.1% formic acid). A nano-scale reverse-phase HPLC capillary column was created by packing 2.6 µm C18 spherical silica beads into a fused silica capillary (100 µm inner diameter x ~30 cm length) with a flame-drawn tip^68^. After equilibrating the column each sample was loaded via a Famos auto sampler (LC Packings) onto the column. A gradient was formed and peptides were eluted with increasing concentrations of solvent B (97.5% acetonitrile and 0.1% formic acid).

As peptides eluted, they were subjected to electrospray ionization and then entered into an LTQ Orbitrap Velos Pro ion-trap mass spectrometer (Thermo Fisher). Peptides were detected, isolated, and fragmented by collision-induced dissociation to produce a tandem mass spectrum of specific fragment ions for each peptide. Peptide sequences (and hence protein identity) were determined by matching protein databases with the acquired fragmentation pattern by the software program, Sequest (Thermo Fisher)^69^. All databases include a reversed version of all the sequences and the data was filtered to between a one and two percent peptide false discovery rate.

### Small and mRNA library preparation

Small RNAs (18 to 30-nt) were size selected on denaturing 15% polyacrylamide gels (Bio-Rad 3450091) from total RNA samples. Small RNAs were treated with 5′ RNA polyphosphatase (Epicenter RP8092H) and ligated to 3′ pre-adenylated adapter with Truncated T4 RNA ligase (NEB M0373L). Small RNAs were then hybridized to the reverse transcription primer, ligated to the 5′ adapter with T4 RNA ligase (NEB M0204L), and reverse transcribed with Superscript III (Thermo Fisher 18080-051). Small RNA libraries were amplified using Q5 High-Fidelity DNA polymerase (NEB M0491L) and size selected on a 10% polyacrylamide gel (Bio-Rad 3450051).

Library concentration was determined using the Qubit 1X dsDNA HS Assay kit (Thermo Fisher Q33231) and quality was assessed using the Agilent BioAnalyzer. Libraries were sequenced on the Illumina NextSeq500 (SE 75-bp reads) platform. For mRNA sequencing, total RNA samples were submitted in triplicate to Novogene Genome Sequencing Company for library preparation. Libraries were sequenced on the Illumina (PE 150-bp reads) platform.

### Clustal Omega alignments and IUPred disorder prediction

Clustal Omega alignment was performed using protein sequences for CSR-1 orthologs available on Wormbase (www.wormbase.org)^70^. When more than one CSR-1 ortholog was present in a given species, a single protein sequence was selected for analysis. Proteins are *C. brenneri* CBN29996 (WBGene00191961), *C. briggsae* CSR-1 (WBGene00037276), *C. elegans* CSR-1, isoform a (WBGene00017641), *C. japonica* CSR-1 (WBGene00126657), *C. latens* PRJNA248912_FL83_15994, *C. nigoni* CSR-1 (PRJNA384657_Cni-csr-1), *C. sinica* PRJNA194557_Csp5_scaffold_00781.g15416.t2, *C. remanei* PRJNA248911_FL82_23103, and *C. tropicalis* PRJNA53597_Csp11.Scaffold629.g12789.t1. For *C. japonica*, exon 1 of CSR-1A was manually annotated from the CJA07453.1 transcript. For disorder prediction, we used IUPred2A (https://iupred2a.elte.hu/) with long disorder parameters and the same protein sequences as were used for Clustal Omega alignment.

### Bioinformatic analysis

For small RNA libraries, sequences were parsed from adapters using FASTQ/A Clipper (options: -Q33 -l 17 -c -n -a TGGAATTCTCGGGTGCCAAGG) and quality filtered using the FASTQ Quality Filter (options: -Q33 -q 27 -p 65) from the FASTX-Toolkit v. 0.0.13 (http://hannonlab.cshl.edu/fastx_toolkit/), mapped to the *C. elegans* genome WS258 using Bowtie2 v. 2.2.2 (default parameters)^71^, and reads were assigned to genomic features using featureCounts (options: -t exon -g gene_id -O --fraction --largestOverlap) which is part of the Subread v. 1.5.1 package^72,73^. Differential expression analysis was performed using DESeq2 v. 1.22.2^74^. To define gene lists from IP experiments, a twofold-change cutoff, a DESeq2 adjusted *p* value of ≤0.05, and at least 10 RPM in the IP libraries were required to identify genes with significant changes in small RNA levels. Additionally, any genes identified as having differentially enriched small RNAs from control samples (HA or FLAG immunoprecipitations from wild-type animals), were removed from further analysis.

For mRNA libraries, sequences were parsed from adapters using Trimmomatic v. 0.36 (options: PE -phred33 ILLUMINACLIP: < fasta with adapter sequences > :2:30:10 LEADING:3 TRAILING:3 SLIDINGWINDOW:4:30 MINLEN:30)^75^ and mapped to the *C. elegans* genome WS258 using HISAT2 v. 2.1.0 (options: --dta-cufflinks --known-splicesite-infile <path to file of known splice sites > )^76^. Reads were assigned to transcripts using featureCounts (options: -t exon -g gene_id -p) which is part of the Subread v. 1.5.1 package^72,73^. Differential expression analysis was performed using DESeq2 v. 1.22.2^74^.

CSR-1 target genes, male CSR-1 target genes, ALG-3/4 target genes, *mutator* target genes, germline-enriched genes, spermatogenesis-enriched genes, and oogenesis-enriched genes were previously described^28,33–40^. Additional data analysis was done using R, Excel, and Python. Venn diagrams were generated using BioVenn^77^ and modified in Adobe Illustrator. Sequencing data is summarized in Supplementary Data 6.

### Reporting summary

Further information on research design is available in the Nature Research Reporting Summary linked to this article.

## Supplementary information

Supplementary Information

Supplementary Data 1

Supplementary Data 2

Supplementary Data 3

Supplementary Data 4

Supplementary Data 5

Supplementary Data 6

Supplementary Data 7

Description of Additional Supplementary Files

Reporting Summary

## Data Availability

The RNA sequencing data generated in this study are available through Gene Expression Omnibus (GEO) under accession code GSE151828. The mass spectrometry proteomics data generated in this study are available through the ProteomeXchange Consortium via the PRIDE partner repository^78^ with the dataset identifier PXD021227. Source data are provided with this paper.

## Source data

Source Data
