# Supplementary material for: Arginine methylation promotes siRNA-binding specificity for a spermatogenesis-specific isoform of the Argonaute protein CSR-1
Source: Nat Commun. 2021 Jul 9;12:4212. doi: 10.1038/s41467-021-24526-6 (PMC8270938; doi:10.1038/s41467-021-24526-6)
Supplement: Supplementary file 1 — Supplementary Information [file 41467_2021_24526_MOESM1_ESM.pdf]

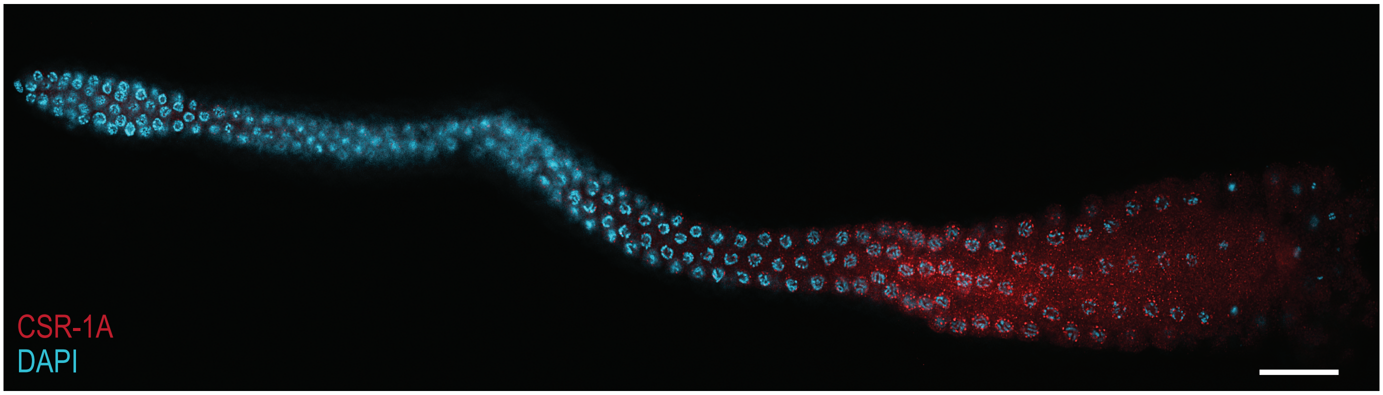

Supplementary Fig. 1 CSR-1A is expressed in the spermatogenesis region of the male germline. Immunofluorescence staining of CSR-1A in a dissected L4 male germline carrying the 2xHA::mCherry::CSR-1A transgene. HA antibodies are used to recognize CSR-1A and DNA is stained with DAPI. The experiment has been reproduced with at least five individual germlines imaged. Scale bar, 20μM.

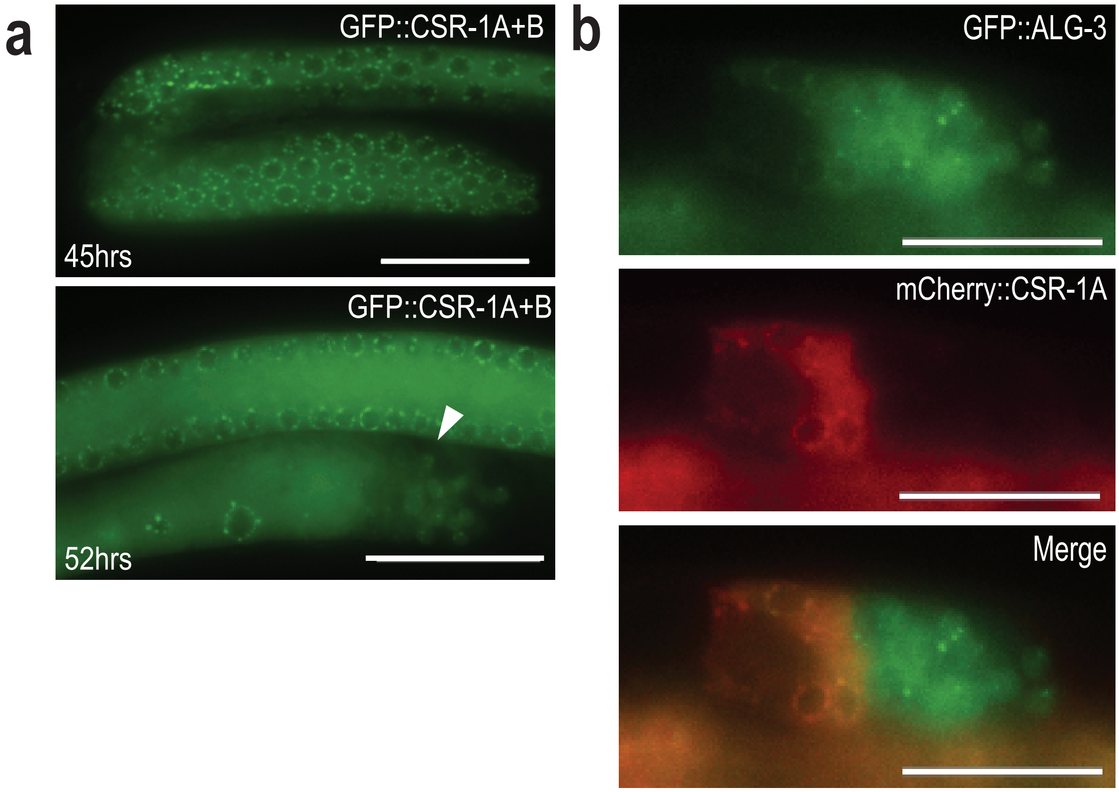

Supplementary Fig. 2 CSR-1A is excluded from secondary spermatocytes.

a 3xFLAG::GFP::CSR-1A+B at 45 hrs (early L4 stage) or 52 hrs (young adult stage) post-L1 arrest. White arrowhead indicates region of secondary spermatocytes. Scale bars, 25μM. **b** Live imaging of double-transgenic animal labelled for both GFP::ALG-3 and mCherry::CSR-1A at the young adult stage. Scale bars, 25μM. At least 5 individual germlines were imaged for each strain and condition.

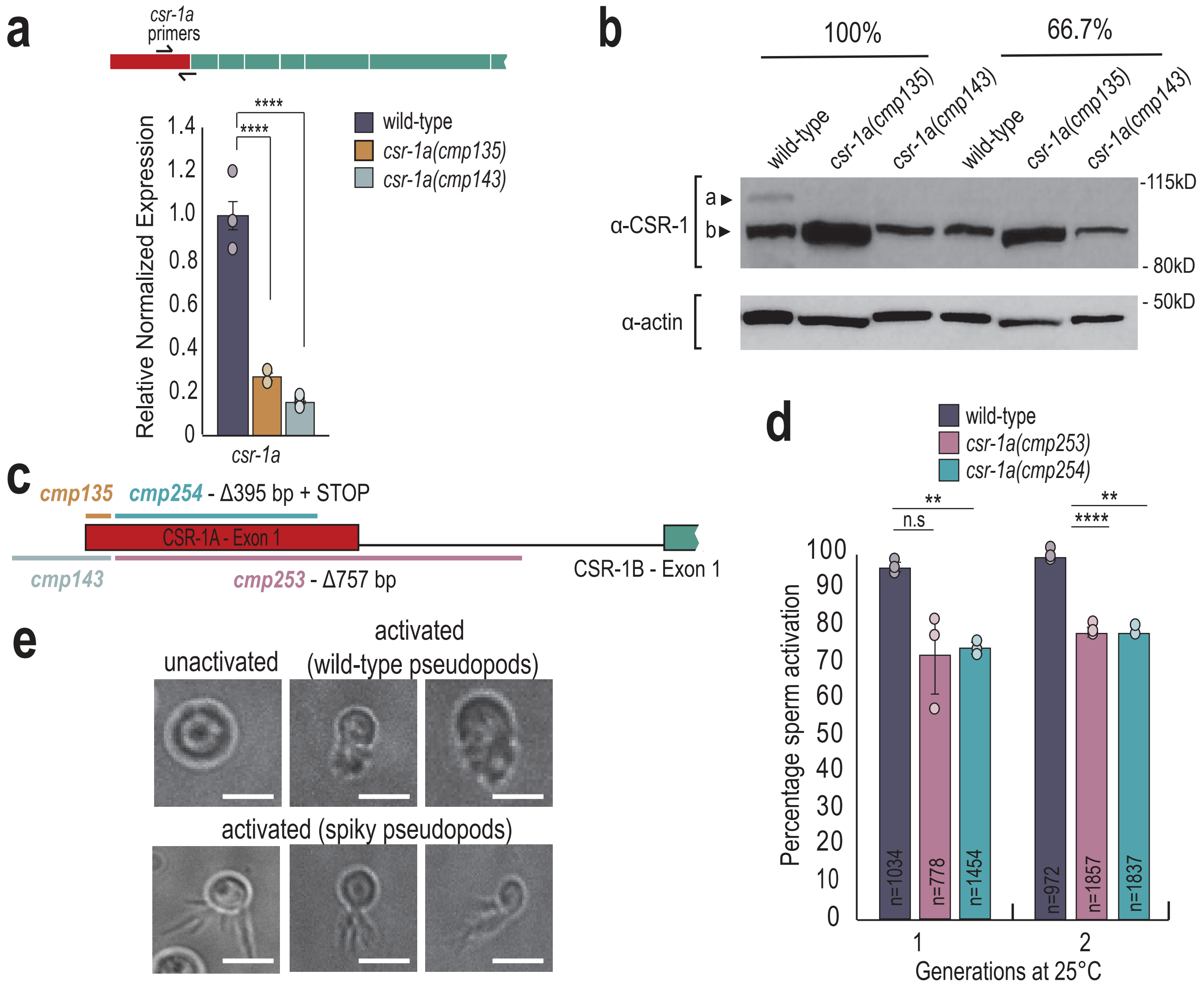

Supplementary Fig. 3 CSR-1A is required for optimal male fertility.

a RT-qPCR on *csr-1a* mutants and wild-type animals at L4. Relative expression was normalized to *rpl-32* and calculated relative to wild-type. Bar graphs representing mean for three biological replicates and error bars indicate SEM. Two-tail t-tests were performed to determine statistical significance. **b** Western blot detecting for both CSR-1 isoforms expression in wild-type and *csr-1a* mutants L4 hermaphrodites at 100% and 66.7% loading volumes, using CSR-1 antibody. Actin is shown as a loading control. The blot has been reproduced. **c** Schematic representation of two new *csr-1a* mutants (*cmp253* and *cmp254*). **d** *in vitro* sperm activation assay on additional *csr-1a* alleles and wild-type. Animals were raised at 25°C for either one or two generations and at least 150 spermatids were counted for each replicate at each generation. Three biological replicates are shown, with bar graphs representing the mean and error bars indicating standard deviation. Two-tail t-tests were performed to determine statistical significance. **e** Representative images from the *in vitro* sperm activation assay of *csr-1b(cmp258)* mutants. Unactivated spermatids and activated spermatids with wild-type pseudopods were observed in wild-type and all mutant strains, while activated spermatids with spiky pseudopods were observed only in *csr-1b(cmp258)* mutants. The phenotype was observed in in all replicates. Scale bars, 5μM. n.s. denotes not significant and indicates a p-value > 0.05, ** indicates a p-value ≤ 0.01, and **** indicates a p-value ≤ 0.0001. See Supplementary Data 7 for more details regarding statistical analysis. Source data are provided as a Source Data file.

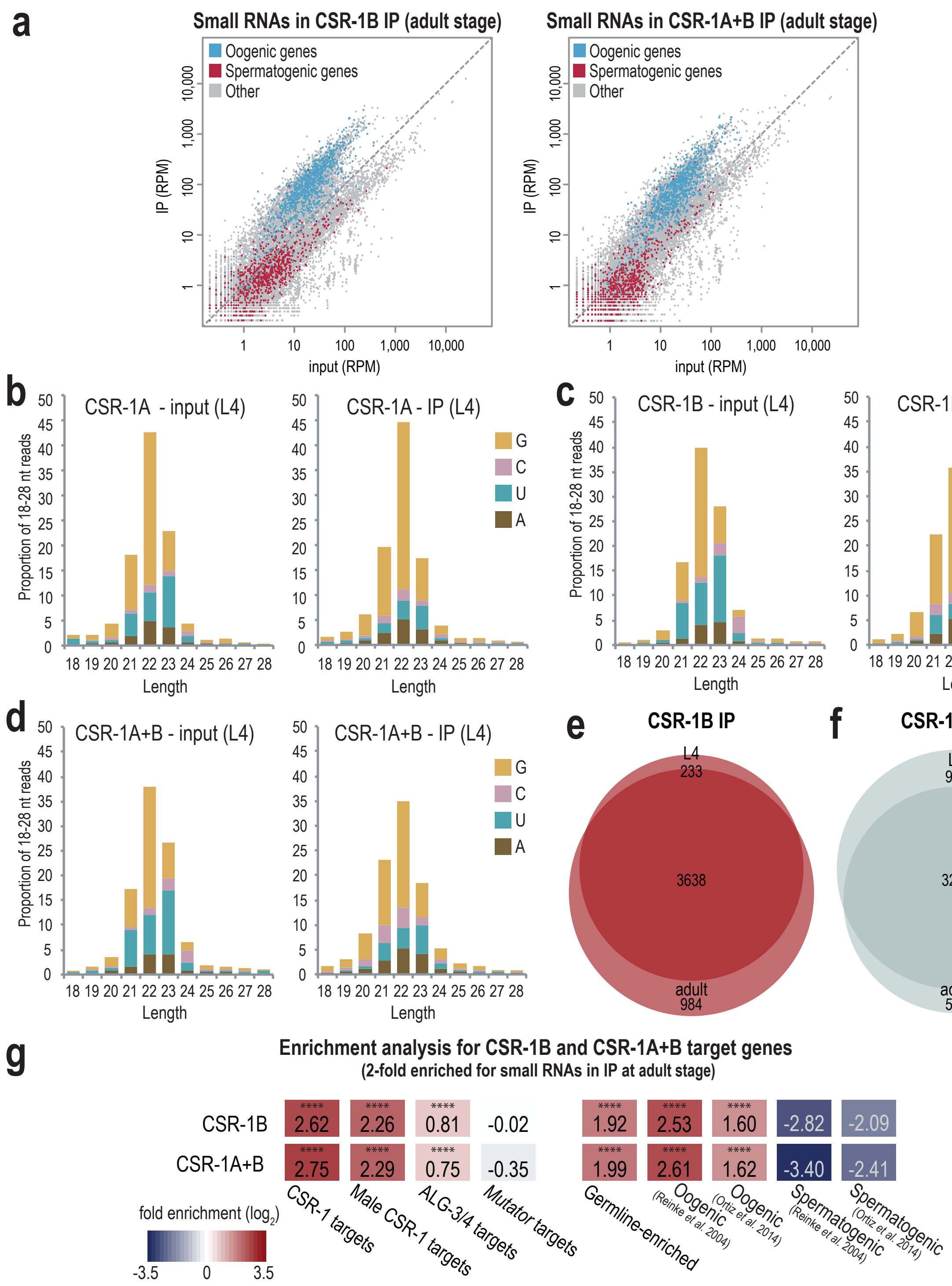

Supplementary Fig. 4 Both CSR-1 isoforms bind germline-enriched 22G-RNAs.
a Normalized reads for oogenic and spermatogenic genes (defined by Reinke *et al*, 2004) at adult stage from FLAG::CSR-1B IP and FLAG::CSR-1A+B IP compared to input. Oogenesis and spermatogenesis genes are indicated in blue and red respectively. **b-d** 5' length and nucleotide distribution in representative input and IP libraries from L4 stage HA::CSR-1A (**b**), FLAG::CSR-1B (**c**), and FLAG::CSR-1A+B (**d**). **e-f** Venn diagram indicates overlap of L4 and adult target genes from CSR-1B (**e**) and CSR-1A+B (**f**) immunoprecipitations. Target genes are defined are at least 2-fold enriched in the IP, with at least 10 RPM in IP samples and a DESeq2 adjusted p-value ≤ 0.05. **g** Enrichment analysis (log2(fold enrichment)) examining the overlap of adult stage CSR-1B and CSR-1A+B target genes with known targets of the CSR-1, male CSR-1, ALG-3/4, and *mutator* small RNA pathways and oogenesis and spermatogenesis-enriched genes. See Materials and Methods for gene list information. Two-tailed p-values for enrichment was calculated using the Fisher's Exact Test function in R. n.s. denotes not significant and indicates a p-value > 0.05 and **** indicates a p-value ≤ 0.0001. See Supplementary Data 7 for more details regarding statistical analysis.

```
Cbn-CSR-1  MQSGSSYNGRGGGGRGRGGGGQD---RGQ---RDGGRDGRGSHRGGRGGGQARPNPYTNNSG-----------------------------RGGRGGGNNQEFSNRGREGSGSYRGGRGG 85
Cjp-CSR-1  MEAQNWNQGRGDGR-GRGGRGRG-----------NGNGNGGSTRGGRGGGQDGE------------------------------------------QGDGSVNGDSRRGGRGGYRGRDGE 65
Cel-CSR-1  MQSDNVGRGRGSRG-GSR-----------------GGSGRGRGRGGYGDSYRPDLETRETHVTYQGKVKYEGGDRHDFNYEKTPTGSY--------RGSHRDGQEGSRGNRGSFRGGRGG 94
Ctr-CSR-1  MQSGNSNRGRDTR---------------GNDRGNSGRGGRGSTRGKRGGGQHQELKVGENSEPGGGRQ------KDHSNAVRGGRGSYRGRGGGLD---QQDQNHGGHGARGSNRGGRGG 96
Cre-CSR-1  MQSENSNRGGRGGG-GR---GRGARDREDRDPGGSGRGGRGSYRGGRGGSERG--------------------------------------GSQ-E---HRFSNRGGRGGRGSHR-GGSE 73
Cla-CSR-1  MQSENSNRGGRGGG-GRG-RGRGARDRDDRDPGGSGRGGRGSYRGGRGGSERG--------------------------------------GSQ-E---HRFSNRGGRGGRGSHR-GGSE 75
Csi-CSR-1  MQSEHSSRGRGVGG-SRG-RGRDYGESS-----NRGRGDRGFSRGGRGGSDRG--------------------------------------RGGFDNGYDREQSRSGRGGRGSYRGGGPE 75
Cbr-CSR-1  MQSENRG--RGG-G-GRG-R---------------GRGDRESGRGAG---------------------------------------------------------------RGSYRDGGSE 37
Cni-CSR-1  MQSEYRG--RGG-G-GRG-R---------------GRGDRESGRGGGAGRG------------------------------------------------SDRGGQGGRGGRGSHRDGGSE 52
           *::                                * ..    **                                                                  *. *

Cbn-CSR-1  YDRGNVPQDQQPS--------------------------FGGQDGRGGYGGRGGNTGYSRGQSNRGNDVRSGRDANYNNDREGSYR--GRGGGGHRGDRGLDNHSRPVTSNERCNGLLPN 177
Cjp-CSR-1  SFR----------------------------------------GRGGGYRGGGSGDG--------------------------NRER-GGAGGSRSYRGGSDNHDHPDSKFGNGTGMLPV 118
Cel-CSR-1  YDRRRDDEES-----KA---------ENRFGGYNRG-------ANGGNYRGRTGGSG-------------------------------------RGGSTQ------TTTVDPKYNGLILE 150
Ctr-CSR-1  YNQVPQQDQR-----QNNDQGERSSHRGGRGAPDHS-PRRGQYQNSGGRGGRGGIAQAPRGRTND-----------FRG-EHGRSSR-GRGNSSRGGSNIVDNHSRPVETNERHNGMLSS 197
Cre-CSR-1  --NGPPSSYRSDRESNGDQRGGRGSHRGDYKGSERGSHHRDDNASRGGYRGRGGHRG---------------------D-GDGAYTRGGG--SDRGRRDHADNHRRPVETTDRCNGLLSS 167
Cla-CSR-1  --NGPPSSYRSDRESNGDQRGGSGSHRGDYKGSERGSHHRD-NASRGGYRGRGGHRG---------------------D-GDGAYTRGGG--SDRGRRDHADNHRRPVETTDRCNGLLSS 168
Csi-CSR-1  --RGPPSNYRDEREENSRGRGGRGR-----GGSNRGSYNNDGNNFRGGYRGRGGTRG---------------------S-SDRDCQR-GGN-NNRERTDYIDNHSRTVEPVGRCNGLLPS 164
Cbr-CSR-1  --RGPPSSSRDERESNNRGRGGY-R-----GGSDRGTSSREGNESRGGYRGRGGPRN---------------------G-QNSSYRGRGGDGHRGGRSNNDDNHARPVEAVGRCNGLLPD 127
Cni-CSR-1  --RGPPSSSREE--YNNRGRGGY-R-----GGSERGNSSREGNESRGGYRGRGGPRD---------------------G-QNSSYRKRGGDGHRGGRSNNDDNHARPVEAVGRCNGLLPD 140
             .                                           *.   *  .                                                          . .*::

Cbn-CSR-1  ---KNENMGDPSDMNQNSNPRLAINIFGLELSERRVFRHVVQSRLLDNKTKKDYILTTMSSRGRGNRASKQKDNFILLGKLLDEYAASKK-EKKHPIFAYDGAQSLFSLEGISMVCEQNN 293
Cjp-CSR-1  -----EGVSDNGEMNQNQTPRLTINIFGLELENRTIFRHVIQIKLLDPKTKKDYILTTMSARGRGNRASKQGDNFILLGKLIDKWVEKTEG--KRPIFAYDGAQTLFALEGISLLLTIPN 231
Cel-CSR-1  NSAEPKLKGLNSDMNQKQNPRLALNIFGLELSERTIFRHVVQMKLIDRQHNKEYILTTMSARGRGNRATKQKDNFILLDILLKQWAAKKG-QQNLPAFAYDGAQSLFTLEGISLMVDIKK 269
Ctr-CSR-1  ---NKENMDNPSKMNSNGNPRLAINIFGLELSERKIFRHVVQMKLLDNKTKKDHILTTMSAKGRGNRASKQKDNFLLLKMLLDKWAAKRG-SNNTPVFAYDGAQSLFTLEGISERMVIKK 313
Cre-CSR-1  ---DKENSGNR-DMNQHLNPRLALNIFGLELSERIVFRHVVQMKLLDHKTNKDYILTTMSARGRGNRASKQNDNFILLGKLLDRWITTKG-TKKHPVYAYDGAQSLFTLEGISQNMVIQK 282
Cla-CSR-1  ---DKENSGNR-DMNQHLNPRLALNIFGLELSERIVFRHVVQMKLLDHKTNKDYILTTMSARGRGNRASKQKDNFILLGKLLDRWITAKG-TKKHPVYAYDGAQSLFTLEGISQNMVIQK 283
Csi-CSR-1  ---EVQNEANRSNMNQNLNPRLALNIFGLELSDRTVFRHVVQMKLLDPRTKKDYILTTMSARGRGNRASKQKDNFILLGKILEKWASRKPGRKSLPIYAYDGAQSLFTLEGISEMLIIKK 281
Cbr-CSR-1  ---DKENKGNSSNMNQN-NPRLAINIFGLELSDRTVFRHVVQMKLLDHKTKKDYILTTMSARGRGNRASKQKDNFILLRKLLDKWAGRKGGKKTDVLFAYDGAQSLFTLEGISELMVIKK 243
Cni-CSR-1  ---DKENKGNSSNMNQN-NPRLAINIFGLELSDRTVFRHVVQMKLLDHKTKKDYILTTMSARGRGNRASKQKDNFILLRKLLDKWAGRKGGKKTDVVFAYDGAQSLFTLEGISELMVIKK 256
                :      .**.: .***::*******.:* :****:* :*:* : :*::******::******:** ***:**  ::..:        .    :******:**:*****       :

Cbn-CSR-1  C--LYL------------------FFFCRPSKSRRKMLFKSPGFQSNVFNCKS--VFVHYFQREIFVTIQRSLRQHHAYGGTPREMGRRCERNTQRMPNRRKHSPIANLGARSY---VYS 388
Cjp-CSR-1  EEAMEIANISHFLKDSIQFLNGDLEISCEPDLD-------KPSFRQTEINEWSDPRFYGYL---DIVTSQSAIRSGR-Y----LSQSKGLYVNTQHMEKLRVGWAVAAKGVHKG----CR 332
Cel-CSR-1  EDALEIPELSNFLKDSISFLSGDLEISCEPDLE-------KPSFVQTELNEWSDPRFYAYL---DIVTSQSAIRSER-Y----LSQSKGLYVHTQSLEELRVKWAVAAKGIHKG----CR 370
Ctr-CSR-1  DDALQIADISEFLKDSVKFLSGDLEISCEPDHD-------KPFFVQTEIREWSDPRFYAYL---DIVTSQGAIRSGK-Y----LSQSKGLYVNTSHMTELRAKWAVAAQGIHKG----CR 414
Cre-CSR-1  EDALQIPGISEFLKDSIKFLNGDLEISCEPDLE-------KPSFVQTEINEWSDPRFYAYL---DIVTSQSATRSGK-Y----LSQSKGLYVNTEHMEELRVKWAVAAKGIHKG----CR 383
Cla-CSR-1  EDALQIPGISEFLKDSIKFLNGDLEISCEPDLE-------KPSFVQTEINEWSDPRFYAYL---DIVTSQSATRSGK-Y----LSQSKGLYVNTEHMEELRVKWAVAAKGIHKG----CR 384
Csi-CSR-1  DEALLIPDISDFLKDSIKYLNGDLEISCEPDLE-------KPSFNQTEINEWSDPRFYAYL---DIVTSQSAIRSGK-Y----LSQSKGLYVNTGHMEELRXXTAFNDRPPRRESTKAVE 386
Cbr-CSR-1  EDALEIPEISEFLKDSIKFLNGDLEITCEPDLE-------KPSFNQTEVNEWSDPRFYAYL---DIVTSQSAIRSGK-Y----LSQSKGLYVNTGHMEELRVKWAVAAKGIHKG----CR 344
Cni-CSR-1  EEALEIPGISEFLKDSIKFLNGDLEITCEPDLE-------KPSFNQTEVNEWSDPRFYAYL---DIVTSQSAIRSGK-Y----LSQSKGLYVNTGHMEELRVKWAVAAKGIHKG----CR 357
              : :                     : *.*. .       .* *  .. ..  *   *  *:    :** * : *. : *     . .:    :*  : : *     .     :

Cbn-CSR-1  IVCC-----DHVKFLAQSTQYYAPIPLNLMIQYAFPRDFPSNR-LVSPNMRLQRAVKLLLKDLKCNPYYEDLQDWATNTITVTDVDYNAPKDSEYRQKYPNLKFPNLPAALCGMGQHKRP 502
Cjp-CSR-1  VVGTQGPLP-ILELDPQSTQYYAPILLNEMLLYAFPREFQSNRPLTNPNQRLQRGVKLLLKDLKCNPYYEDKGSFATNTIIISDIDYNAHRDQEYRDRYPKIQFPHLPAAVCGMGPRRRL 451
Cel-CSR-1  IVGANGPLP-ILELDPQSTQYYASIPLSQMLQYAFPRDFPPNR-IVNPNMKLQRAVKLLLKDLKCNPYYDDKQIWATNTITVSDVDYNAPKDPEFRQKYPNLKFPMLPAVQCGTGPHKRL 488
Ctr-CSR-1  IVGSTGPLP-VLELDPQSTQYYAPIPLSQMIQLAFPRDFNANR-GVNPNMRLQRAVKLLLKDLKCNPFYEDLRDWATNTITVSDIDYNAHKDNDYKLKYPNLRFPYLPAAQCGTGPSRRL 532
Cre-CSR-1  IVGKEHPLP-ILELDPQSTQYYAAIPLSKMIQYAFPRDFGSNR-IVNPNLRLQRAVKLLLKDLKCNPYYEDLSNWATNTITVSDIDYNAPKNQEFKQKYPNLKFPALPAAQCGTGPHKRL 501
Cla-CSR-1  IVGKEHPLP-ILELDPQSTQYYAAIPLSKMIQYAFPRDFGSNR-IVNPNMRLQRAVKLLLKDLKCNPYYEDLSNWATNTMTVSDVDYNAPKNQEFKQKYPNLKFPALPAAQCGTGPHKRL 502
Csi-CSR-1  LLALTTRFQFWSSTVTQSTQYYAPIPLNQMLLYAFPRDFAPNR-MVNPNMRLQRQVKLLLKDLKCNPFYEDLADWGTNTNTVSDVDFNAPKDAEYRQKHPNLKFPLLPAAQCGHGPNKRL 505
Cbr-CSR-1  IVGTGHPLP-ILELDPQSTQYYAAIPLSQMIQYAFPRDFVPNR-MINPNMRLQRAVKLLLKDLKCNPFYEDLADWATNTITVSDVDYNAPKDPEYRQKYPNLRFPNFPAAQCGNGPHRRL 462
Cni-CSR-1  IVGTGHPLP-ILELDPQSTQYYAAIPLSQMIQYAFPRDFVPNR-MINPNMRLQRAVKLLLKDLKCNPFYEDLADWATNTITVSDVDYNAPKDPEYRQKYPNLKFPNFPAAQCGNGPHRRL 475
           ::          .    ******* * *. *:  ****:*  **    .** :*** ************:*:*    :.***  ::*:*:** :: ::: ::*:::** :**. ** *  :*

Cbn-CSR-1  MPLEYLRVLPYQSIDRRVLEEFELTPRANAPNERWSTLQQHFDEFGFNDEVMQLFGVQICNDPFQNLSEIDGERVTAPKIAYADPVHVDDDKRDWKAQDKKFAKAATIDHLVFVLVAGYT 622
Cjp-CSR-1  MPLEYLRVLPFQSIDRRVLEEFELTPRANAPEERWKTLQSHFDEFGFNDEVMQAFGVEICNDPFNNVSEIDGARIDKPSIAYADPVHVDDEKRDWKAQDKKFVAPAKIDYLLFVLVAGYT 571
Cel-CSR-1  MPLEYLKVLPYQSIDRRVLEEFELTPRANAPNERWSTLQKHYDQFGFNDQVMKDFGVQICNDPFNNVSEIDGERVLAPSVAYADPVHVDDEKRDWKAQDKKFVTPATIDHLMFVLVAGYT 608
Ctr-CSR-1  MPLEYLKVLPYQSIDRRVLEEFELTPRANAPNERWQILQKHYDEFGFNNEVMIEFGVLICNDPFQNISEIDGERVLAPKISYADPVHVDDEKRDWKAQDKKFAKAAVIDHLVFVLVAGYT 652
Cre-CSR-1  MPLEYLKVLPFQSIDRRVLEEFELTPRANAPNERWTTLQHHYDAFGFNDSVMQEFGVLICNDPFQNISEIDGERVLAPKIAYADPVHVDDEKRDWKAQDKRFETPATIDHLMFVLLAGYT 621
Cla-CSR-1  MPLEYLKVLPFQSIDRRVLEEFELTPRANAPNERWTTLQHHYDAFGFNDDVMQEFGVLICNDPFQNISEIDGERVLAPKIAYADPVHVDDEKRDWKAQDKRFETPATIDHLMFVLLAGYT 622
Csi-CSR-1  VPLEHLKVLPFQSIDRRVLEEFELTPRANAPNERWTILQHHYEEFGFNDKVMQDFGVLICNDPFQNISEIDGERVVAPKINYADPVHVDDEKRDWKAQDKKFESPATIDHLVFVLVAGYS 625
Cbr-CSR-1  MPLEYLKVLPFQSVDRRVLEEFELTPRANAPNERWTTLQHHYEEFGFNDQVMQEFGVQICNDPFQNISEIDGERVLAPKITYADPVHVDDEKRDWKAQDKKFETPATIHHLVFVLVAGYS 582
Cni-CSR-1  MPLEYLKVLPFQSVDRRVLEEFELTPRANAPNERWTTLQHHYEEFGFNDQVMQEFGVQICNDPFQNISEIDGERVLAPKITYADPVHVDDEKRDWKAQDKKFETPATIHHLVFVLVAGYS 595
           :***:*:***:**:****************:***  ** *:: ****:.**  *** ******:*:***** *:  *.: *********:*********:*   * *.:*:***:***:

Cbn-CSR-1  RNFEADIKATEYVARSFMQRCNDKGMVIRNYEFRSWEGERESEHFLTRVFKDLVTHPKYRDSSFIPFVLLVSDDVPNIHECLKFEERMSDIPTQHILLKNIRKIRDNIEKKSQGGRRGYD 742
Cjp-CSR-1  RDFKGDTMATEYVARAFMQRCREKGISLGNFDFYSHQGERDSENFLTSVFKRLVTHPKYHDSNFTPFVVFVSDDVPNIHECLKFEERMSDIPTQHILLKNVRKIRDNIVRKSAGGRRAYD 691
Cel-CSR-1  RTWDADCDATKFVAKAFMQRCKDKGMHIGSYSMDQHNGERGSENFLTSVFKNLVTHPNYRDSSFTPFVLFISDDVPNIHECLKFEERMSDIPTQHVLLKNVKKMRDNIEKKSQGGRRAYD 728
Ctr-CSR-1  RNFKDDIRATEYVARAFMQRCQDKGMTIRNYELQTHEGERDSEAFLTSVFKKLVTHPKYRDSSFIPFVLFVSDDVPNIHECLKFEERMSDIPTQHVLLKNIRKIRDNMERKSGGGRRAYD 772
Cre-CSR-1  RSFDQDIRATEFVANAFMQRCRDKGMIIKNYELKHFEGERDSETFLTSVFKYLVTLREYPNPAFIPFVLFVSDDVPNIHECLKFEERMSDIPTQHVLLKNIRKIRDNIEKKSHGGRRAYD 741
Cla-CSR-1  RSFDQDIRATEFVANAFMQRCRDKGMIIKNYELKHFEGERDSETFLTSVFKYLVTLREYPNPAFIPFVLFVSDDVPNIHECLKFEERMSDIPTQHVLLKNIRKIRDNIEKKSHGGRRAYD 742
Csi-CSR-1  RHFDSEIKASEFVARAFMQRCADKGMRICNYEMRHHEGERDSENFLTSVFKNLVTHPKYRDPSFIPFVLFISDDVPNIHECLKFEERMSDIPTQHVLLKNIRKIRDNIEKKSQGGRRAYD 745
Cbr-CSR-1  RNFDAEIKATEFVARAFMQRCNDKGMRIDNYELRSFEGERNSENFLTSVFKNLVTHNRYRDPSFIPFVLFVSDDVPNIHECLKFEERMSDIPTQHILLKNIRKIRDNIEKKSQGGRRAYD 702
Cni-CSR-1  RNFEAEIKATEFVARAFMQRCNDKGMRIDNYELRSFEGERNSENFLTSVFKNLVTHNRYRDPSFIPFVLFVSDDVPNIHECLKFEERMSDIPTQHILLKNIRKIRDNIEKKSQGGRRAYD 715
           * :. :  *:::**.:***** :**: : .:.:   :*** ** *** *** ***  .* :  * ***::***********************:****::*:***: :** ****.**

Cbn-CSR-1  LTLDNIVMKANIKCGGLNYTADVPRDLACWRDTPTFVMGMDVAHPDKTSVREGNPSTVGVSCLSCNSADHPYSFIGDFLFTDPRQEAIQDQILRKFTDQNVRNFAEIRGFPKKIIIFRDG 862
Cjp-CSR-1  LTLDNIVMKANVKCGGLNYTADIPQDIACWRDVSTFVMGMDVAHPDKAASREGNPSTVGL---SCNSADNPYTFIGDFHYTDPRQETIQDEILRRFTDHSVRNFAEFRGFPKKIIIFRDG 808
Cel-CSR-1  LTLDNIVMKANIKCGGLNYTADIPRDLACWNEVSTFVIGMDVAHPDRNAAREGNPSTVGL---SCNSAENPYSFIGDFLYTDPRREAIQDEILRKFTDQSVRNFAEIRGFPKKVIIFRDG 845
Ctr-CSR-1  LTLDNIVMKANIKCGGLNYTADIPRDIACWNDTPTFVMGMDVAHPDRLATREGNPSTVGL---SCNAAEHPYSFIGDFLYTDPRREAIQDEILRKFTDQNVRNFAEIRGFPKKIIIFRDG 889
Cre-CSR-1  LTLDNIVMKANVKCGGLNYTADPPRELGAWKEVPTFVMGMDVAHPDKMASREGNPSTVGL---SCNAADNPYSFIGDFLYTDPRREAIQDELLRKFTDQHVRNFAERRGFPKKIIIFRDG 858
Cla-CSR-1  LTLDNIVMKANVKCGGLNYTADPPKDLGAWKEVPTFVMGMDVAHPDKMASREGNPSTVGL---SCNAADNPYSFIGDFLYTDPRREAIQDELLRKFTDQHVRNFAERRGFPKKIIIFRDG 859
Csi-CSR-1  LTLDNIVMKANVKCGGLNYTADVPRDIGSWREVPTFVMGMDVAHPDKMASREGNPSTVGL---SCNSAESPYSFIGDFLYTDPRREAIQDEILRKFTDQNVRNFADRRGFPKRIIIFRDG 862
Cbr-CSR-1  LTLDNIVMKANVKCGGLNYTADIPRDIGSWREVSTFVMGMDVAHPDKMATREGSPSTVGL---SCNSAESPYSFVGDFLYTDPRREAIQDEILRKFTDQNVRNFAERRGFPKRIIIFRDG 819
Cni-CSR-1  LTLDNIVMKANVKCGGLNYTADIPRDIGSWREVSTFVMGMDVAHPDKMATREGSPSTVGL---SCNSAESPYSFVGDFLYTDPRREAIQDEILRKFTDQNVRNFAERRGFPKRIIIFRDG 832
           ***********:********** *:::..*.:. ***:********: : ***.*****:    ***:*: **:*:*** :****:*:***::**:***: *****: *****::******

Cbn-CSR-1  VSFGEEKEAMREVEIIEQTIISAAKAMGLRDYAPKVLAIVVKKRHHTRFYARGGNQGDTPINPLPDTSVGGDIAEYGKRQIFIQAFRPVQGTAKVPSFLIIRDDEELTDEHITRMVCAVC 982
Cjp-CSR-1  VSSGEENAAFKEVGIIENTIVSAANSMGLCDYSPKVLAIVVKKRHHTRFYAKGGYRGTQPMNPMPDTSVGGDIAEYGKRQIFIQAFRPVQGTAKVPSFLIIRDDERVSDDHISKLVCAVC 928
Cel-CSR-1  VSFGEETAALKEVEIIEQTIKTAAKSMGHSDYAPKVLAIVVKKRHHTRFYAKGGHHGNMPINPLPDTSVGGDIAEYGKRQIFIQAFRPVQGTAKVPSFLVIRDDEEVSDEHVAKMVCAVC 965
Ctr-CSR-1  VSFGEESEALREVEIIEQTIISAAMSMGLRDYAPKVLAIVVKKRHHTRFYAKGGHNGMTPSNPLPDTSVGGDIAEYGKRQIFIQAFRPVQGTAKVPSFLILRDDEEVSDEHISKMVCAVC 1009
Cre-CSR-1  VSFGEETEALREVEIIEQTIIAAAKSQGLRDYAPKVLAIVVKKRHHTRFYAKGGQNGNTPTNPLPDTSVGGDIAEYGKRQIFIQAFRPVQGTAKVPSFLILRDDEEVSDESIAKMTCAVC 978
Cla-CSR-1  VSFGEETEALREVEIIEQTIIAAAKSQGHRDYAPKVLAIVVKKRHHTRFYAKGGQNGNTPTNPLPDTSVGGDIAEYGKRQIFIQAFRPVQGTAKVPSFLILRDDEEVSDESIAKMTCAVC 979
Csi-CSR-1  VSFGEETEALREVKIIEETCIAAAKSMGHRDYAPKVLAIVVKKRHHTRFYAKGGHHGNMPANPLPDTSVGGDIAEYGKRQIFIQAFRPVQGTAKVPSFLIIRDDEEVTDEHITKMVCAVC 982
Cbr-CSR-1  VSFGEETEALREVKIIEETCIAAAKSMGHRDYAPKVLAIVVKKRHHTRFYAKGGHHGNMPTNPLPDTSVGGEIAEYGKRQIFIQAFRPVQGTAKVPSFLIIRDDEEVTDDHITKMVCAVC 939
Cni-CSR-1  VSFGEETEALREVKIIEETCIAAAKSMGHRDYAPKVLAIVVKKRHHTRFYAKGGHHGNMPTNPLPDTSVGGEIAEYGKRQIFIQAFRPVQGTAKVPSFLIIRDDEEVTDDHITKMVCAVC 952
           ** ***. *::** ***:*  :** : *   **:******************:** .*  * **:*******:*****************************::****.::*: ::::.****

Cbn-CSR-1  SLHQLVNSPTSIPTPVYYVAHELAKRGTGLFK-----CGELRDNWATLTDQLSYSTLDRLSKVRVV 1042
Cjp-CSR-1  SLHQLVNSPTSIPTPVYYVAHELAKRGTGLFKAYRFKRGDIRDDWHTLTNQLSYSTLDRLSKVRVV 993
Cel-CSR-1  SLHQLVNSPTSIPTPVYYVAHELAKRGTGLYKAYRFKNGELFDDWETLTTQLSYSTLDRLSKVRVV 1030
Ctr-CSR-1  SLHQLVNSPTSIPTPVYYVAHELAKRGTGLFKAYRFKNGELRDDWDTLTNQLSYSTLDRLSKVRVV 1074
Cre-CSR-1  SLHQLVNSPTSIPTPVYYVAHELAKRGTGLFKAYRFKNGELH-DWATLSDQLSYSTLDRLSKVRVV 1042
Cla-CSR-1  SLHQLVNSPTSIPTPVYYVAHELAKRGTGLFKAYRFKNGELH-DWATLSDQLSYSTLDRLSKVRVV 1043
Csi-CSR-1  SLHQLVNSPTSIPTPVYYVAHELAKRGTSLFKAYRFKNGEIH-DWCTLSDQLSYSTLDRLSKVRLV 1046
Cbr-CSR-1  SLHQLVNSPTSIPTPVYYVAHEMAKRGTGLFKAYRFKNGELR-DWATLSDQLSYSTLDRLSKVRVV 1003
Cni-CSR-1  SLHQLVNSPTSIPTPVYYVAHEMAKRGTGLFKAYRFKNGELR-DWATLSDQLSYSTLDRLSKVRVV 1016
           *********************:*****.*:*      *::  :* **: **************:*
```

Supplementary Fig. 5 Clustal Omega alignment of CSR-1 in Caenorhabditis species.

Arginine/glycine motifs are highlighted in yellow and start codons for both CSR-1A and CSR-1B are highlighted in blue. See Materials and Methods for protein sequence information.

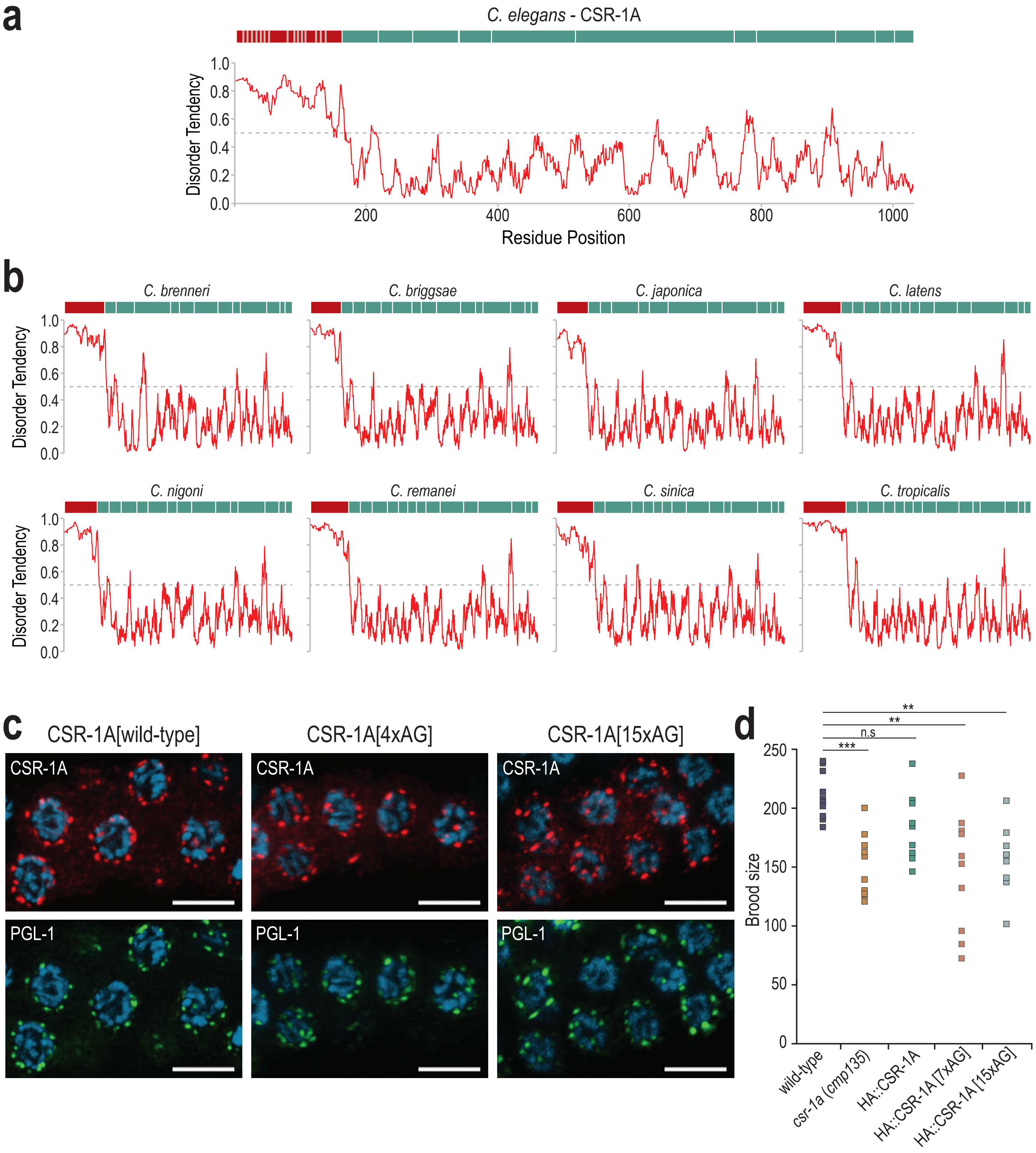

Supplementary Fig. 6 The N-terminal exon of CSR-1 is disordered across Caenorhabditis species.
a-b Graphs examining the disorder tendencies of CSR-1A in C. elegans (**a**) and CSR-1 in related nematode species (**b**). Residue position is plotted on the x-axes and disorder tendency scores (y-axes) above 0.5 indicate disorder. Red bar above each plot marks region of first exon and green bars mark all other exons. **c** Immunofluorescence staining of dissected L4 hermaphrodite germlines in HA::CSR-1A, HA::CSR-1A[4xAG], and HA::CSR-1A[15xAG], using antibodies against HA and PGL-1. Scale bars, 5μM. At least five individual germlines were imaged for each strain. d Brood size assay on wild-type and mutant animals raised at 25°C (n=10). Two-tail t-tests were performed to determined statistical significance. ** indicates p-value ≤ 0.01, *** indicates a p-value ≤ 0.001. See Supplementary Data 7 for more details regarding statistical analysis.
