# Supplementary material for: Arginine methylation promotes siRNA-binding specificity for a spermatogenesis-specific isoform of the Argonaute protein CSR-1
Source: Nat Commun. 2021 Jul 9;12:4212. doi: 10.1038/s41467-021-24526-6 (PMC8270938; doi:10.1038/s41467-021-24526-6)
Supplement: Supplementary file 9 — Description of Additional Supplementary Files [file 41467_2021_24526_MOESM9_ESM.pdf]

Description of Additional Supplementary Files

File Name: **Supplementary Data 1**
Description: Small RNA enrichment in CSR-1 immunoprecipitations.

File Name: **Supplementary Data 2**
Description: mRNA expression in *csr-1a(cmp135)* and *csr-1a(cmp143)* mutant animals.

File Name: **Supplementary Data 3**
Description: Peptides captured from 2xHA::CSR-1A IP-mass spectrometry experiment.

File Name: **Supplementary Data 4**
Description: Reagents, resources, and strains used in this study.

File Name: **Supplementary Data 5**
Description: Oligonucleotides sequences used in this study.

File Name: **Supplementary Data 6**
Description: Sequencing library statistics.

File Name: **Supplementary Data 7**
Description: Summary of statistical analysis.
