# Supplementary material for: Arginine methylation promotes siRNA-binding specificity for a spermatogenesis-specific isoform of the Argonaute protein CSR-1
Source: Nat Commun. 2021 Jul 9;12:4212. doi: 10.1038/s41467-021-24526-6 (PMC8270938; doi:10.1038/s41467-021-24526-6)
Supplement: Supplementary file 10 — Reporting Summary [file 41467_2021_24526_MOESM10_ESM.pdf]

nature research

Corresponding author(s): Carolyn M. Phillips

Last updated by author(s): 6/21/2021

Reporting Summary

Nature Research wishes to improve the reproducibility of the work that we publish. This form provides structure for consistency and transparency in reporting. For further information on Nature Research policies, see our Editorial Policies and the Editorial Policy Checklist.

Statistics

For all statistical analyses, confirm that the following items are present in the figure legend, table legend, main text, or Methods section.

n/a	Confirmed	
☐	☒	The exact sample size (*n*) for each experimental group/condition, given as a discrete number and unit of measurement
☐	☒	A statement on whether measurements were taken from distinct samples or whether the same sample was measured repeatedly
☐	☒	The statistical test(s) used AND whether they are one- or two-sided<br>*Only common tests should be described solely by name; describe more complex techniques in the Methods section.*
☐	☒	A description of all covariates tested
☐	☒	A description of any assumptions or corrections, such as tests of normality and adjustment for multiple comparisons
☐	☒	A full description of the statistical parameters including central tendency (e.g. means) or other basic estimates (e.g. regression coefficient) AND variation (e.g. standard deviation) or associated estimates of uncertainty (e.g. confidence intervals)
☐	☒	For null hypothesis testing, the test statistic (e.g. *F*, *t*, *r*) with confidence intervals, effect sizes, degrees of freedom and *P* value noted<br>*Give P values as exact values whenever suitable.*
☒	☐	For Bayesian analysis, information on the choice of priors and Markov chain Monte Carlo settings
☒	☐	For hierarchical and complex designs, identification of the appropriate level for tests and full reporting of outcomes
☒	☐	Estimates of effect sizes (e.g. Cohen's *d*, Pearson's *r*), indicating how they were calculated

Our web collection on statistics for biologists contains articles on many of the points above.

Software and code

Policy information about availability of computer code

Data collection	No software was used.
Data analysis	For small RNA libraries, sequences were parsed from adapters using FASTQ/A Clipper (options: -Q33 -l 17 -c -n -a TGGAATTCTCGGGTGCCAAGG) and quality filtered using the FASTQ Quality Filter (options: -Q33 -q 27 -p 65) from the FASTX-Toolkit v. 0.0.13 (http://hannonlab.cshl.edu/fastx_toolkit/), mapped to the C. elegans genome WS258 using Bowtie2 v. 2.2.2 (default parameters), and reads were assigned to genomic features using FeatureCounts (options: -t exon -g gene_id -O --fraction –largestOverlap) which is part of the Subread v. 1.5.1 package. Differential expression analysis was done using DESeq2 v. 1.22.2. To define gene lists from IP experiments, a 2-fold-change cutoff, a DESeq2 adjusted p-value of ≤0.05, and at least 10 RPM in the IP libraries were required to identify genes with significant changes in small RNA levels. Additionally, any genes identified as having differentially enriched small RNAs from control samples (HA or FLAG immunoprecipitations from wild-type animals), were removed from further analysis.<br>For mRNA libraries, sequences were parsed from adapters using Trimmomatic v. 0.36 (options: PE -phred33 ILLUMINACLIP:<fasta with adaptor sequences>:2:30:10 LEADING:3 TRAILING:3 SLIDINGWINDOW:4:30 MINLEN:30) and mapped to the C. elegans genome WS258 using HISAT2 v. 2.1.0 (options: --dta-cufflinks --known-splicesite-infile <path to file of known splice sites>). Reads were assigned to transcripts using FeatureCounts (options: -t exon -g gene_id -p) which is part of the Subread v. 1.5.1 package. Differential expression analysis was performed using DESeq2 v. 1.22.2.

For manuscripts utilizing custom algorithms or software that are central to the research but not yet described in published literature, software must be made available to editors and reviewers. We strongly encourage code deposition in a community repository (e.g. GitHub). See the Nature Research guidelines for submitting code & software for further information.

Data

Policy information about availability of data

All manuscripts must include a data availability statement. This statement should provide the following information, where applicable:

- Accession codes, unique identifiers, or web links for publicly available datasets
- A list of figures that have associated raw data
- A description of any restrictions on data availability

The RNA sequencing data generated in this study are available through Gene Expression Omnibus (GEO) under accession code GSE151828 (https://www.ncbi.nlm.nih.gov/geo/query/acc.cgi?acc=GSE151828). The mass spectrometry proteomics data generated in this study are available through the ProteomeXchange Consortium via the PRIDE partner repository78 with the dataset identifier PXD021227 (http://www.ebi.ac.uk/pride/archive/projects/PXD021227).

Field-specific reporting

Please select the one below that is the best fit for your research. If you are not sure, read the appropriate sections before making your selection.

☒ Life sciences ☐ Behavioural & social sciences ☐ Ecological, evolutionary & environmental sciences

For a reference copy of the document with all sections, see nature.com/documents/nr-reporting-summary-flat.pdf

Life sciences study design

All studies must disclose on these points even when the disclosure is negative.

Sample size	small RNA and mRNA seq data analysis: 2-3 biological replicates (see Supplementary Table 6). RT-qPCR analysis: 3 biological and 3 technical replicates. Sample sizes were not predetermined by statistical methods, but by conventional requirements in the respective fields.
Data exclusions	One set of small RNA libraries was excluded because the libraries contained high levels of degraded rRNA. The samples were generated again and new libraries were constructed.
Replication	All immunofluorescence and live imaging experiments were performed at least two times, and at least 3 germlines were imaged per sample, per condition, with similar results. All western blots were performed at least twice. Additional information about number of replicates for specific experiments can be found in the figure legends.
Randomization	For all experiments, control and experimental samples were treated in parallel and animals were chosen randomly from plates for experiments such as brood size and imaging.
Blinding	Blinding was not used in this study. The experiments in this study were not subjective.

Reporting for specific materials, systems and methods

We require information from authors about some types of materials, experimental systems and methods used in many studies. Here, indicate whether each material, system or method listed is relevant to your study. If you are not sure if a list item applies to your research, read the appropriate section before selecting a response.

Materials & experimental systems

n/a	Involved in the study	
☐	☒	Antibodies
☒	☐	Eukaryotic cell lines
☒	☐	Palaeontology and archaeology
☐	☒	Animals and other organisms
☒	☐	Human research participants
☒	☐	Clinical data
☒	☐	Dual use research of concern

Methods

n/a	Involved in the study	
☒	☐	ChIP-seq
☒	☐	Flow cytometry
☒	☐	MRI-based neuroimaging

Antibodies

Antibodies used

Anti-FLAG M2 (Sigma Aldrich F1804, RRID:AB_262044), 1:1,000 Western, 1:500 IF
Anti-HA 3F10 (Roche 11867423001, RRID:AB_390918), 1:500 IF
Anti-HA 3F10 Peroxidase (Roche 12013819001, RRID:AB_390917), 1:1,000 Western
Anti-PGL-1 (DSHB K76, RRID:AB_531836), 1:100 for IF
Anti-CSR-1 (Claycomb Lab), 1:2,000 Western
Anti-actin IgG (Abcam ab3280, RRID:AB_303668), 1:10,000 Western
Goat anti-mouse IgG Alexa Fluor 488 (Thermo Fisher A-11029, RRID:AB_138404), 1:1,000 IF

Goat anti-Rat IgG Alexa Fluor 555 (Thermo Fisher A-21434, RRID:AB_2535855), 1:1,000 IF
Goat anti-mouse IgM Alexa Fluor 647 (Thermo Fisher A21238, RRID:AB_1500930), 1:500 IF
Goat anti-mouse IgG Secondary HRP (Thermo Fisher A16078, RRID:AB_2534751), 1:5,000 Western
Goat anti-rat IgG Secondary HRP (Thermo Fisher A18871, RRID:AB_2535648), 1:5,000 Western
Goat anti-rabbit Secondary HRP (Thermo Fisher A16110, RRID: AB_2534782), 1:5,000 Western

Validation

Anti-PGL-1 has been validated in multiple publications, including Strome and Wood (1983) and anti-CSR-1 has been validated by Claycomb et al (2009). Anti-FLAG and anti-HA have been validated by the manufacturer. Specifically -
Anti-PGL-1 - Validated by Immunofluorescence detecting for a 40 kDa P-granules polypeptide in C. elegans embryos (Strome and Wood 1983)
Anti-CSR-1 - Validated by Immunofluorescence and Western Blot detecting for CSR-1 polypeptide from amino acid E462 to E987 in C. elegans dissected adult germlines and whole cell lysate (Claycomb et al, 2009)
Anti-FLAG M2 - Validated by Western Blot detecting for the N-term FLAG-BAP Fusion Protein in either bacterial, mammalian, or plant extract, then indirectly detected using Anti-Mouse IgG Peroxidase, and visualized using HRP chemiluminescent substrates (https://www.sigmaaldrich.com/deepweb/assets/sigmaaldrich/product/documents/754/849/anti-flag-2poster.pdf)
Anti-HA 3F10 - Validated by Western Blot detecting for purified HA-tagged Glutathione-S-transferase in eukaryotic cell extract, then indirectly detected using Anti-Rat-Ig-Biotin and Streptavidin-POD* using BM Chemiluminescence Western Blotting substrate (POD) (https://www.sigmaaldrich.com/deepweb/assets/sigmaaldrich/product/documents/248/175/roahahabul.pdf)
Anti-HA 3F10 Peroxidase - Validated by Western Blot detecting for purified HA-tagged Glutathione-S-transferase in eukaryotic cell extract, visualized by BM Chemiluminescence Blotting Substrate (POD) (https://www.sigmaaldrich.com/deepweb/assets/sigmaaldrich/product/documents/760/007/12013819001bul.pdf)
Anti-actin IgG - Validated by Western Blot in mammalian cell lines (NIH 3T3, MDA-MB-231, HeLa) whole cell lysates, and mouse liver whole tissue lysate, then indirectly detected by goat anti-mouse IgG polyclonal. Developed by ECL technique (https://www.abcam.com/actin-antibody-actn05-c4-ab3280.html)

Animals and other organisms

Policy information about studies involving animals; ARRIVE guidelines recommended for reporting animal research

Laboratory animals

C. elegans strains (all animals used are hermaphrodites unless otherwise noted in the figure legends):
N2 (wild-type)
GE24
CB151
WM300
USC868
USC896
USC1066
USC988
USC1137
USC1092
USC1139
USC1110
USC1111
USC1112
USC1065
USC1074
USC1258
USC1259
USC1260
USC1159
USC1262
USC1263
USC1264
USC1284
USC1348
USC1326
USC1343
USC1127
USC1128

Wild animals

Wild animals were not used

Field-collected samples

Field-collected samples were not used

Ethics oversight

No ethical approval is required for C. elegans.

Note that full information on the approval of the study protocol must also be provided in the manuscript.
